# Supplementary material for: Establishing a High-Throughput Locomotion Tracking Method for Multiple Biological Assessments in Tetrahymena
Source: Cells. 2022 Jul 28;11(15):2326. doi: 10.3390/cells11152326 (PMC9367449; doi:10.3390/cells11152326)
Supplement: Supplementary file 1 [file cells-11-02326-s001.zip › SOP for tetrahymena tracking.pptx]

## Slide 1
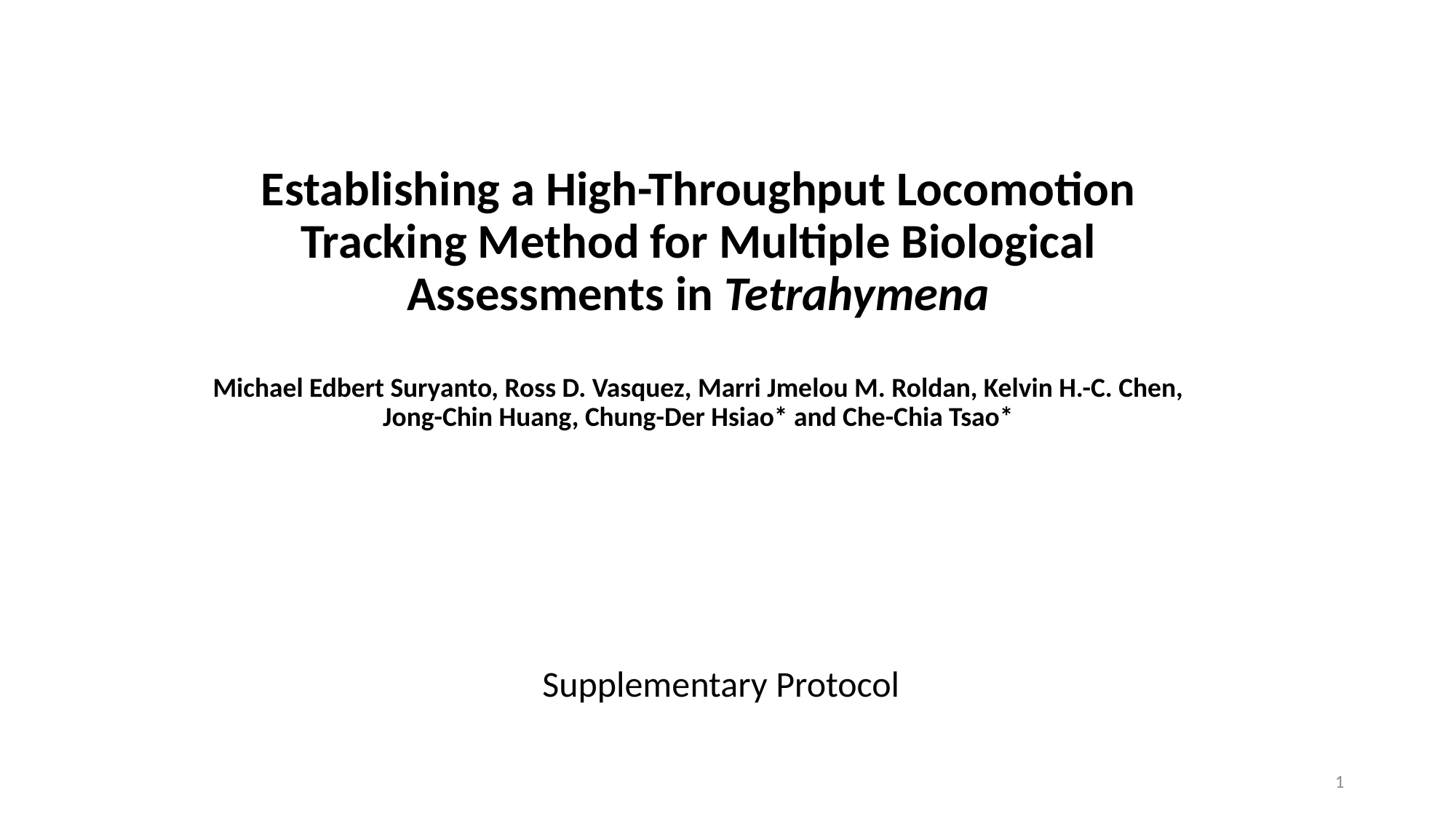

# Establishing a High-Throughput Locomotion Tracking Method for Multiple Biological Assessments in Tetrahymena Michael Edbert Suryanto, Ross D. Vasquez, Marri Jmelou M. Roldan, Kelvin H.-C. Chen, Jong-Chin Huang, Chung-Der Hsiao* and Che-Chia Tsao*
Supplementary Protocol
1

## Slide 2
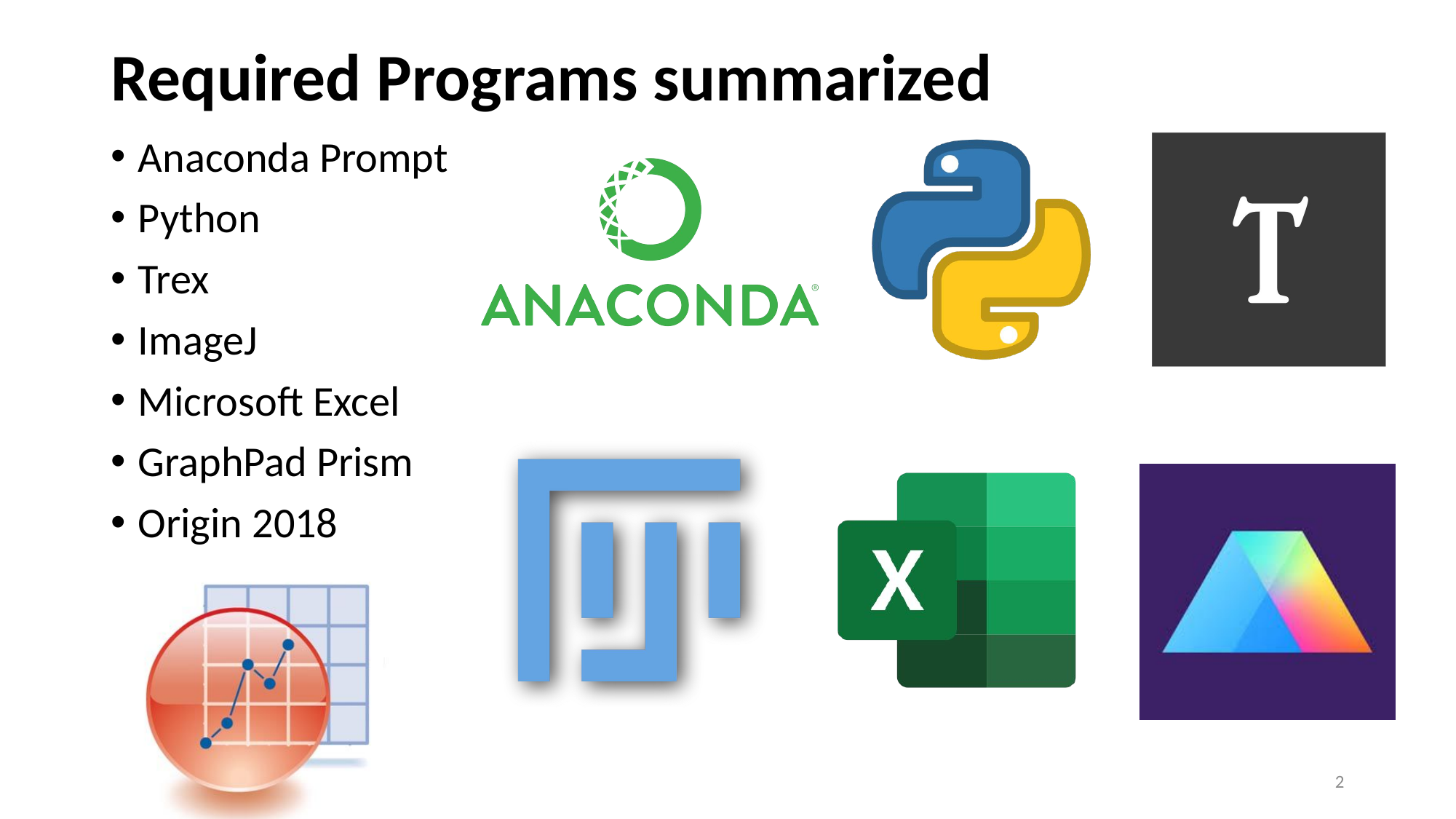

# Required Programs summarized
Anaconda Prompt
Python
Trex
ImageJ
Microsoft Excel
GraphPad Prism
Origin 2018
2

## Slide 3
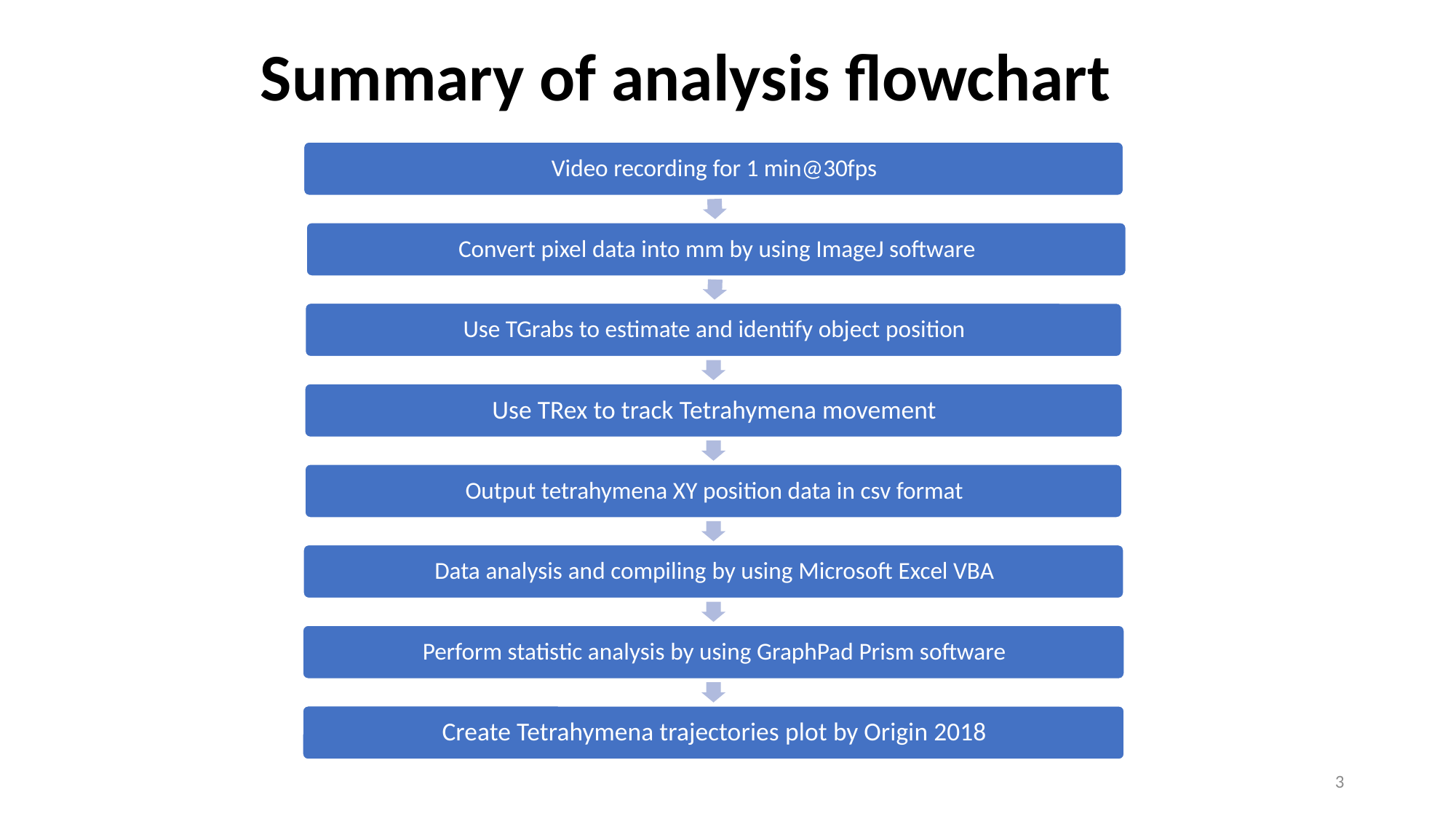

# Summary of analysis flowchart
3

## Slide 4
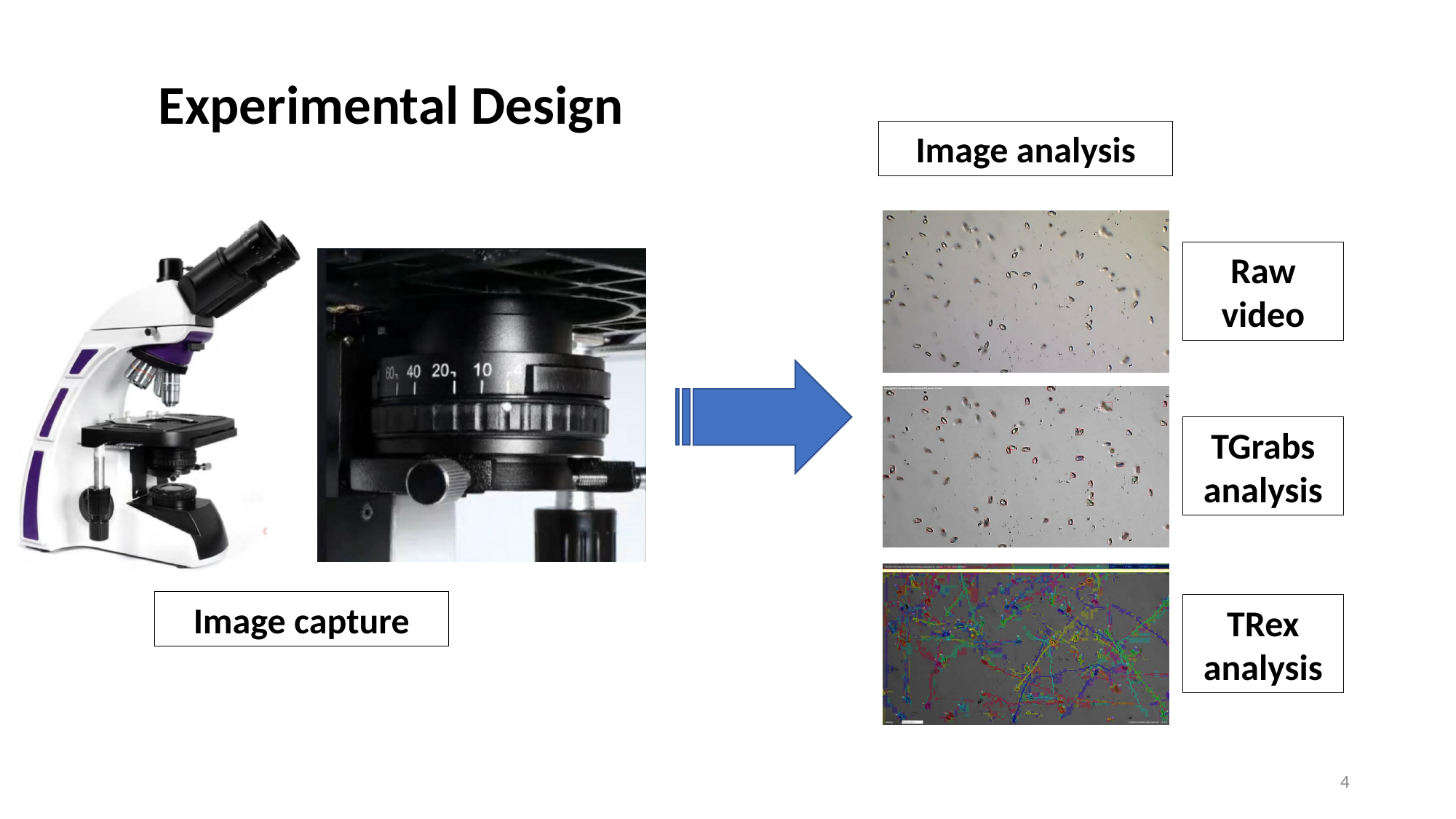

Experimental Design
Image analysis
Raw video
TGrabs analysis
Image capture
TRex analysis
4

## Slide 5
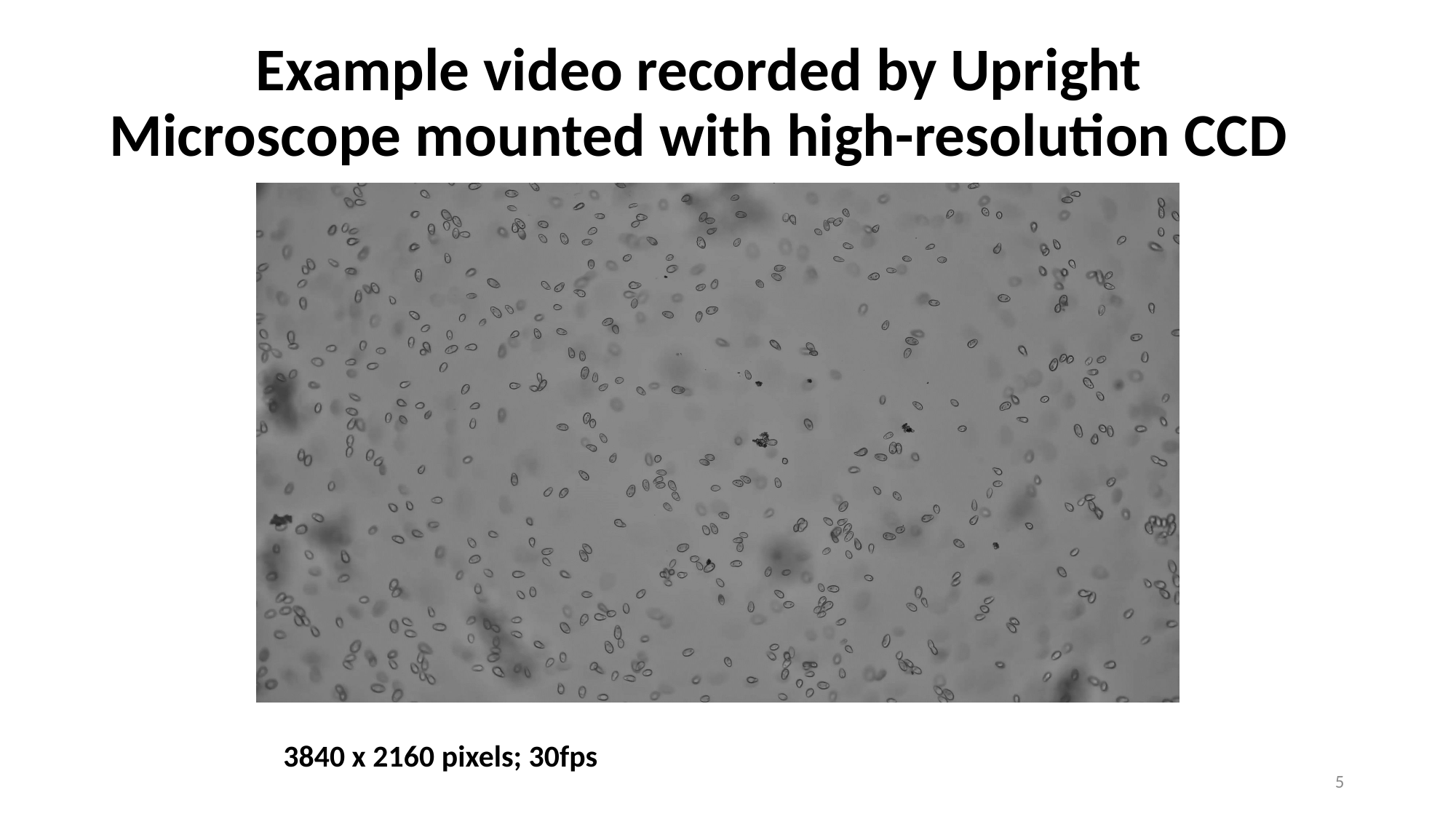

# Example video recorded by Upright Microscope mounted with high-resolution CCD
3840 x 2160 pixels; 30fps
5

## Slide 6
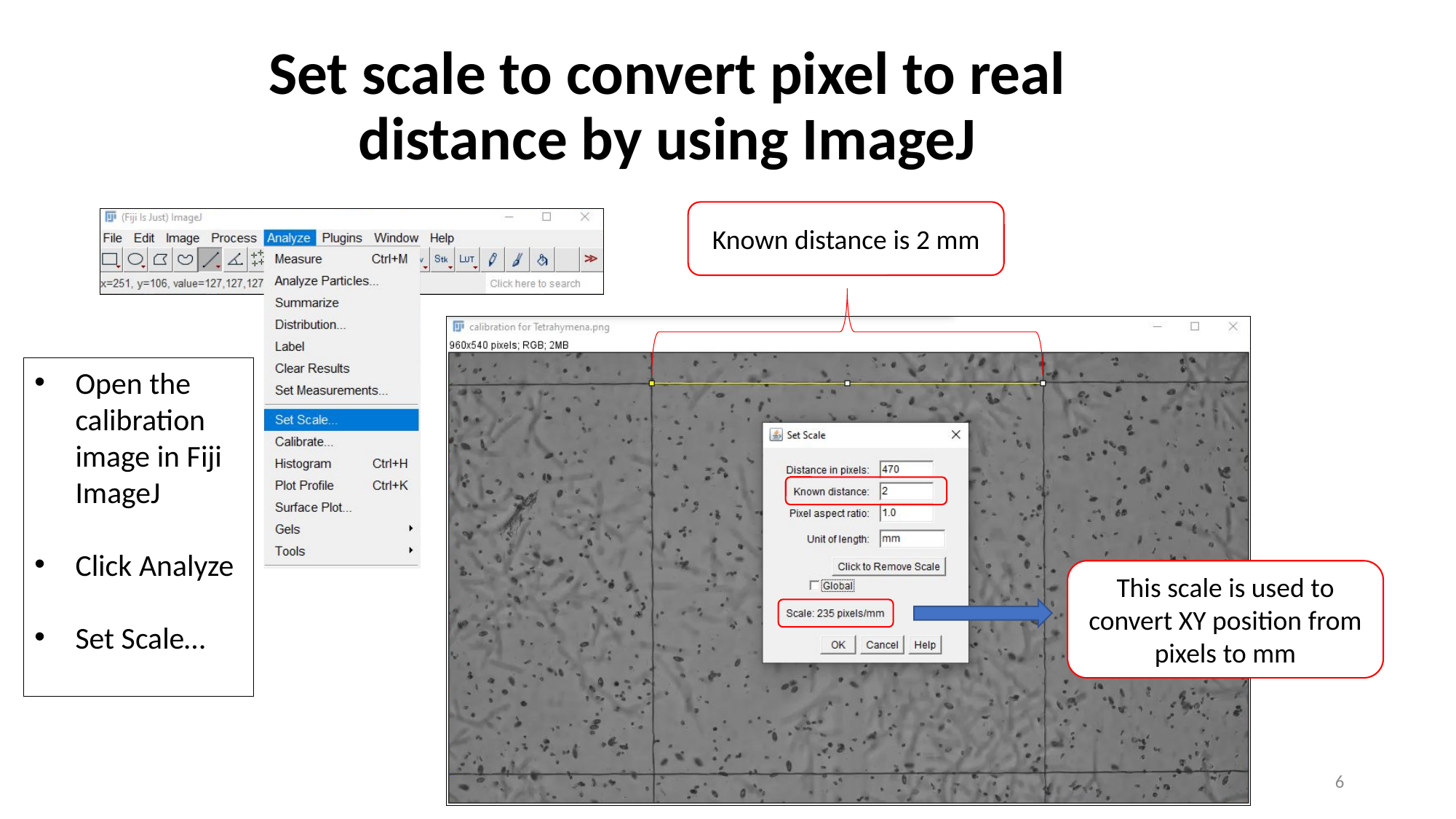

# Set scale to convert pixel to real distance by using ImageJ
Known distance is 2 mm
Open the calibration image in Fiji ImageJ
Click Analyze
Set Scale…
This scale is used to convert XY position from pixels to mm
6

## Slide 7
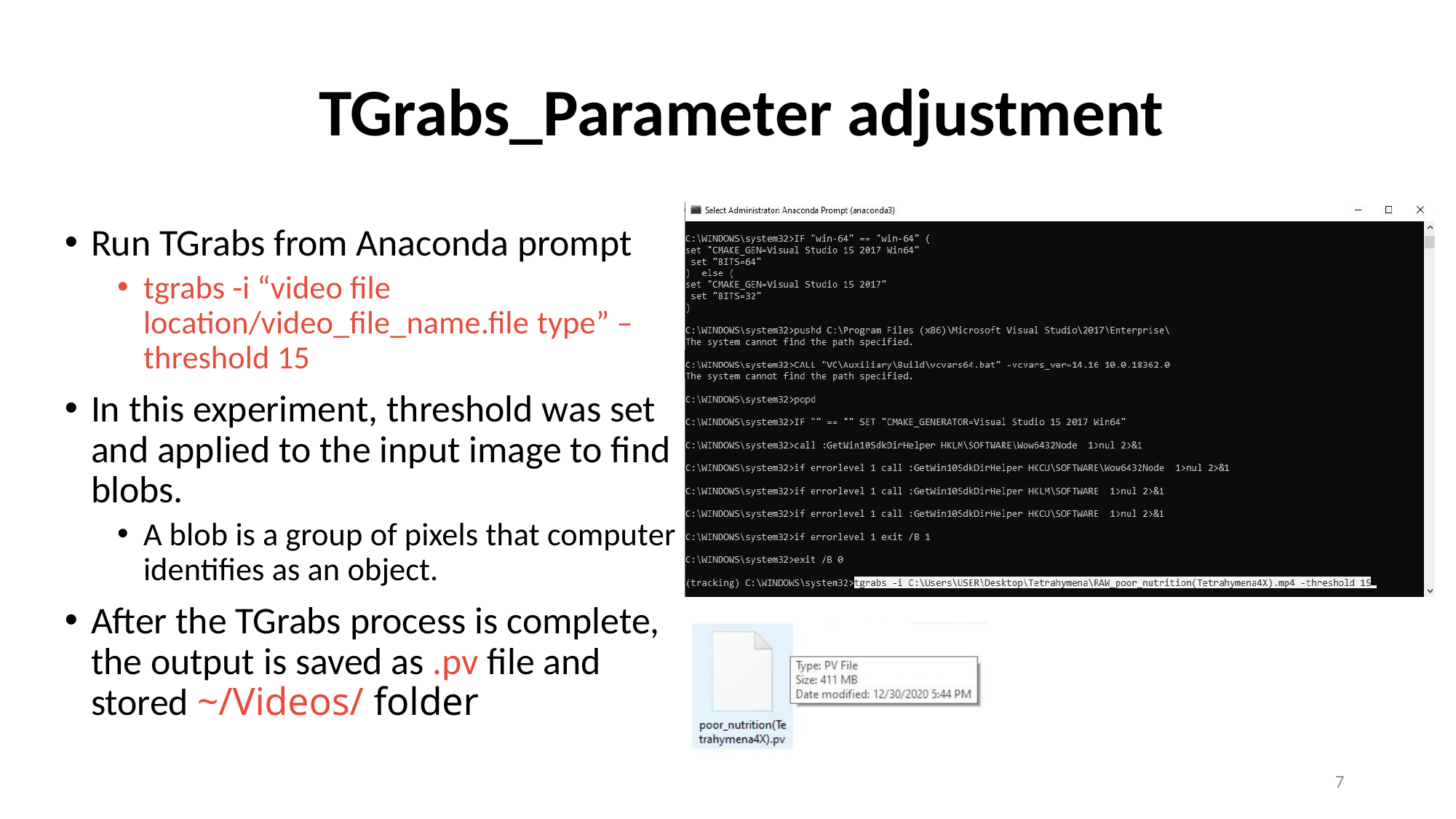

# TGrabs_Parameter adjustment
Run TGrabs from Anaconda prompt
tgrabs -i “video file location/video_file_name.file type” –threshold 15
In this experiment, threshold was set and applied to the input image to find blobs.
A blob is a group of pixels that computer identifies as an object.
After the TGrabs process is complete, the output is saved as .pv file and stored ~/Videos/ folder
7

## Slide 8
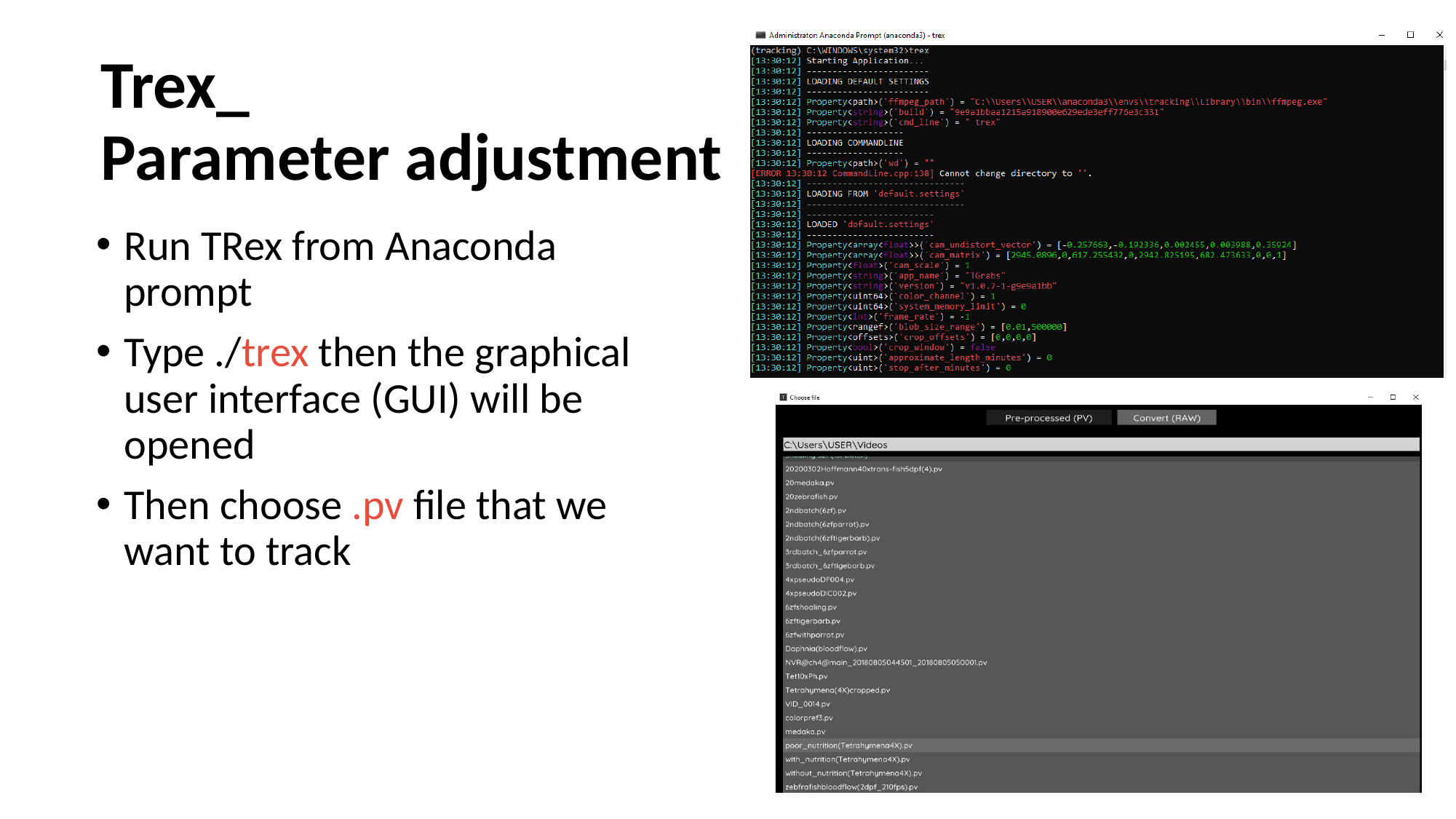

# Trex_ Parameter adjustment
Run TRex from Anaconda prompt
Type ./trex then the graphical user interface (GUI) will be opened
Then choose .pv file that we want to track
8

## Slide 9
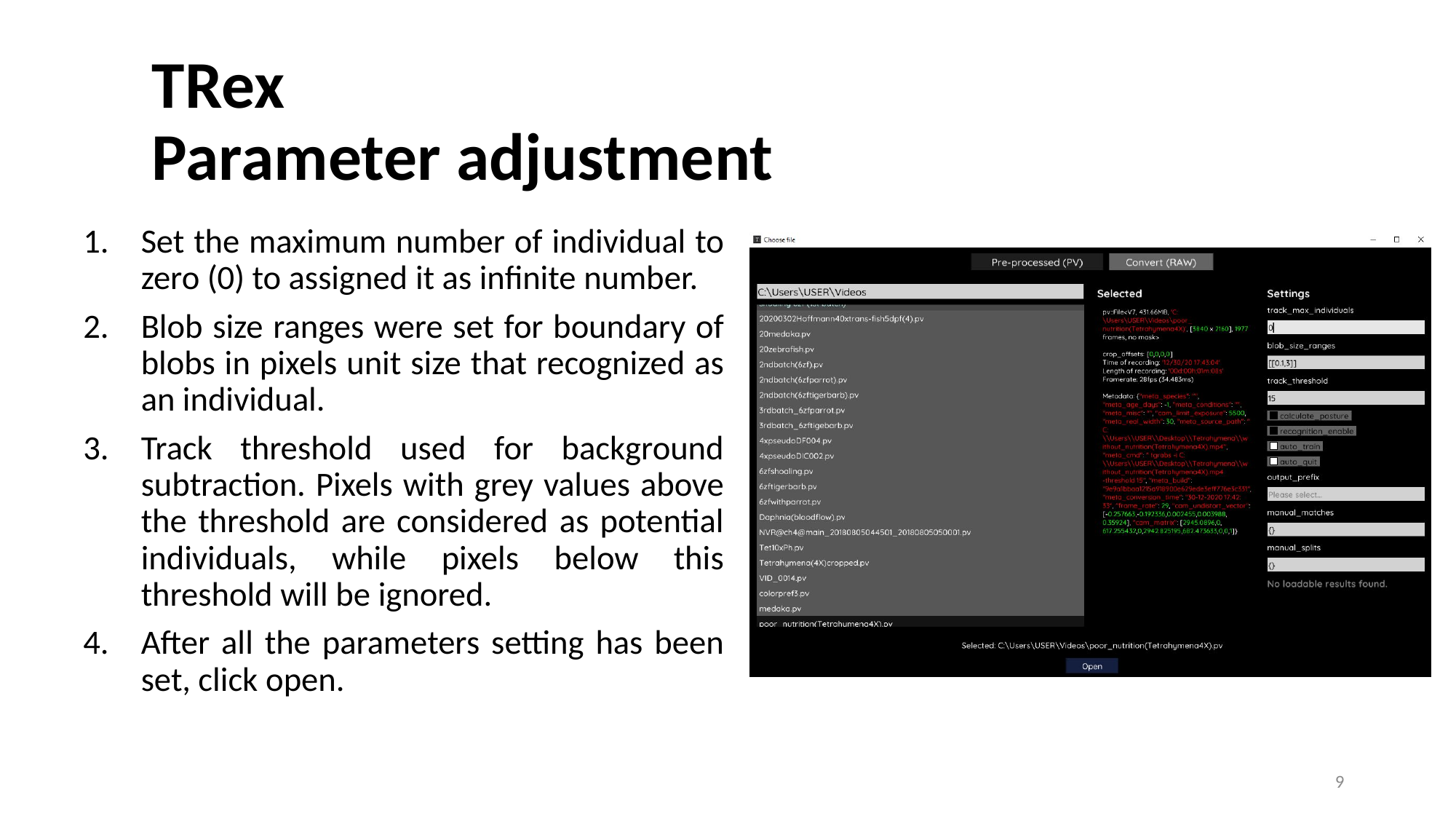

# TRex Parameter adjustment
Set the maximum number of individual to zero (0) to assigned it as infinite number.
Blob size ranges were set for boundary of blobs in pixels unit size that recognized as an individual.
Track threshold used for background subtraction. Pixels with grey values above the threshold are considered as potential individuals, while pixels below this threshold will be ignored.
After all the parameters setting has been set, click open.
9

## Slide 10
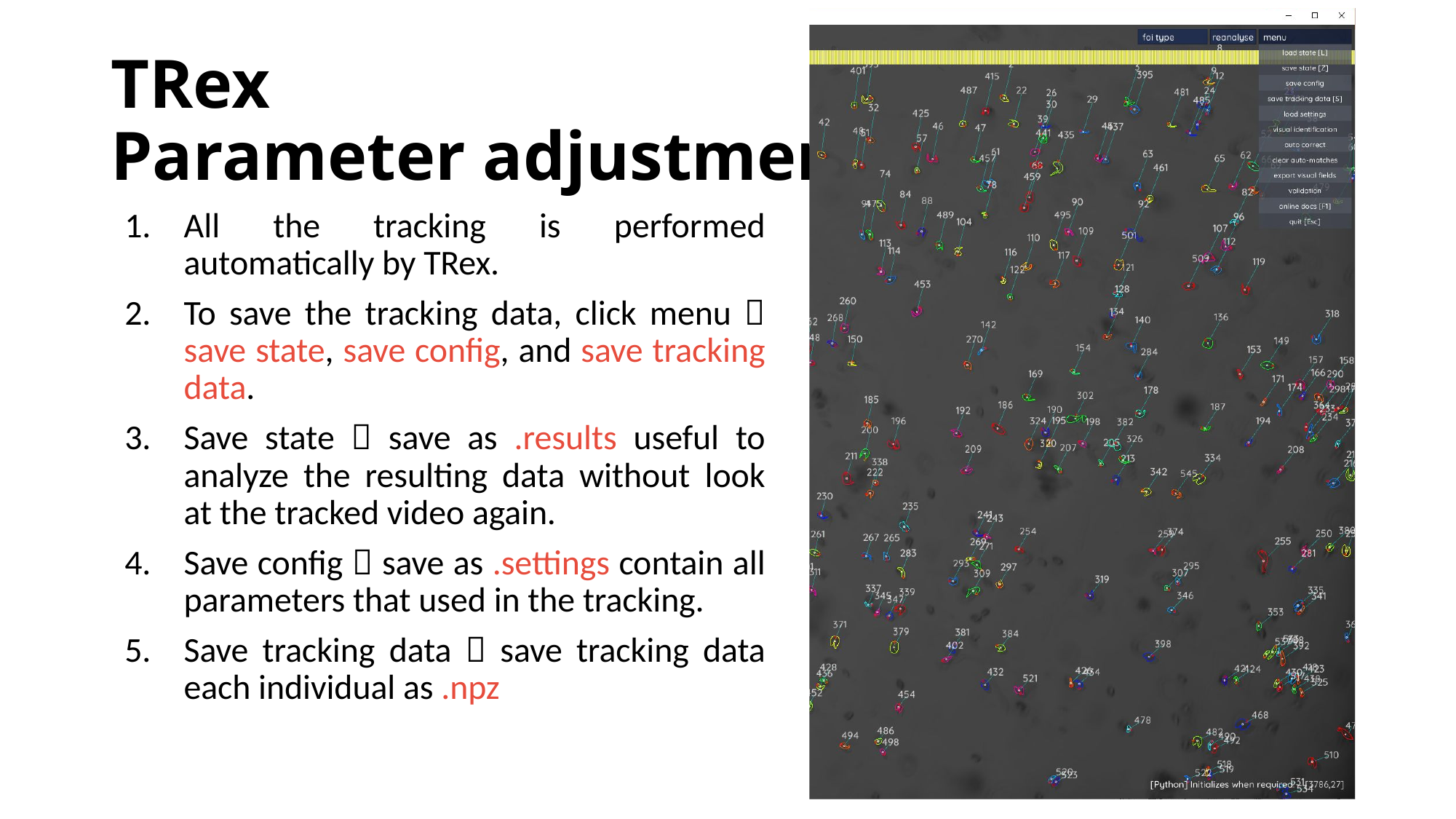

# TRex Parameter adjustment
All the tracking is performed automatically by TRex.
To save the tracking data, click menu  save state, save config, and save tracking data.
Save state  save as .results useful to analyze the resulting data without look at the tracked video again.
Save config  save as .settings contain all parameters that used in the tracking.
Save tracking data  save tracking data each individual as .npz
10

## Slide 11
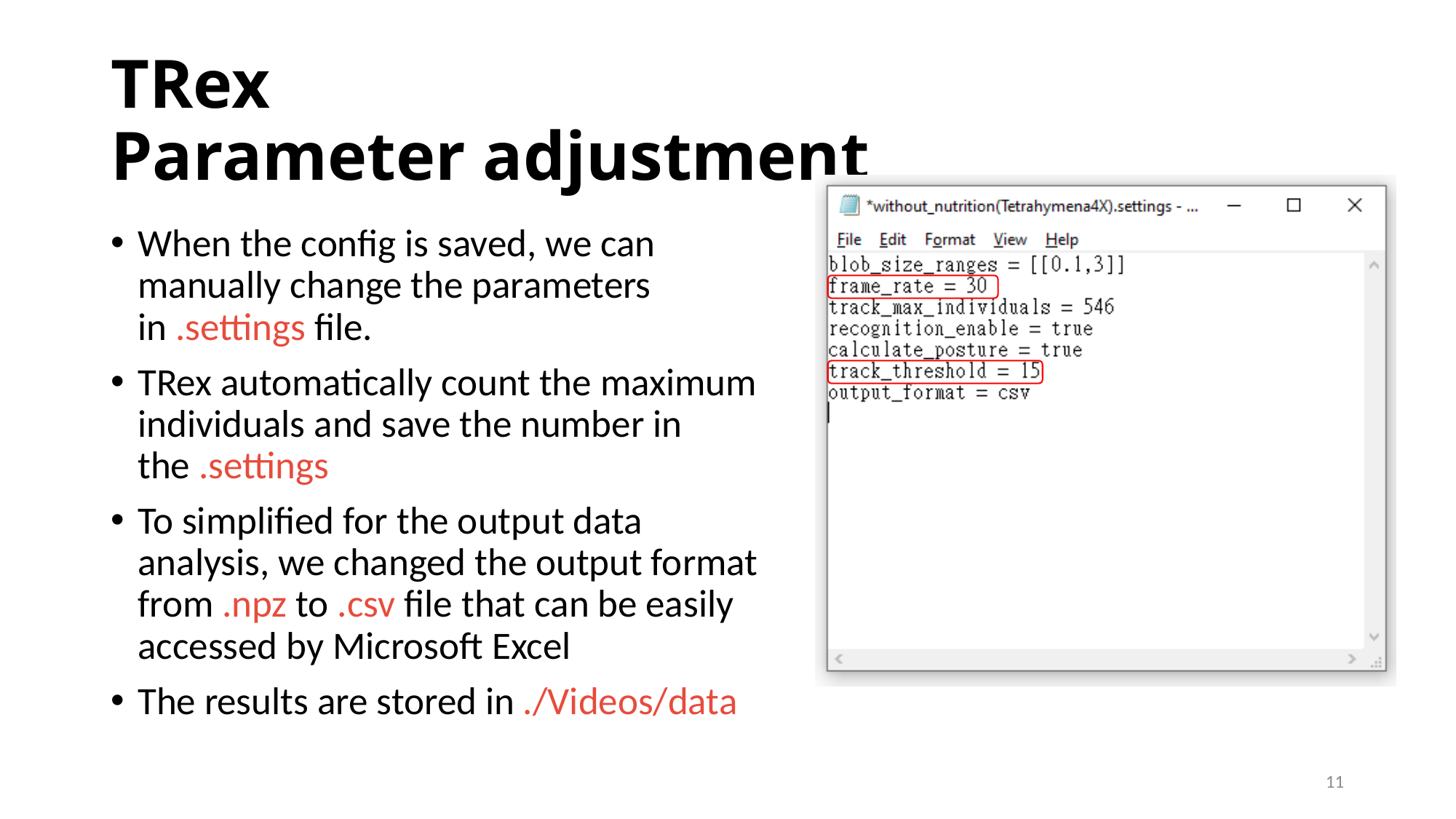

# TRex Parameter adjustment
When the config is saved, we can manually change the parameters in .settings file.
TRex automatically count the maximum individuals and save the number in the .settings
To simplified for the output data analysis, we changed the output format from .npz to .csv file that can be easily accessed by Microsoft Excel
The results are stored in ./Videos/data
11

## Slide 12
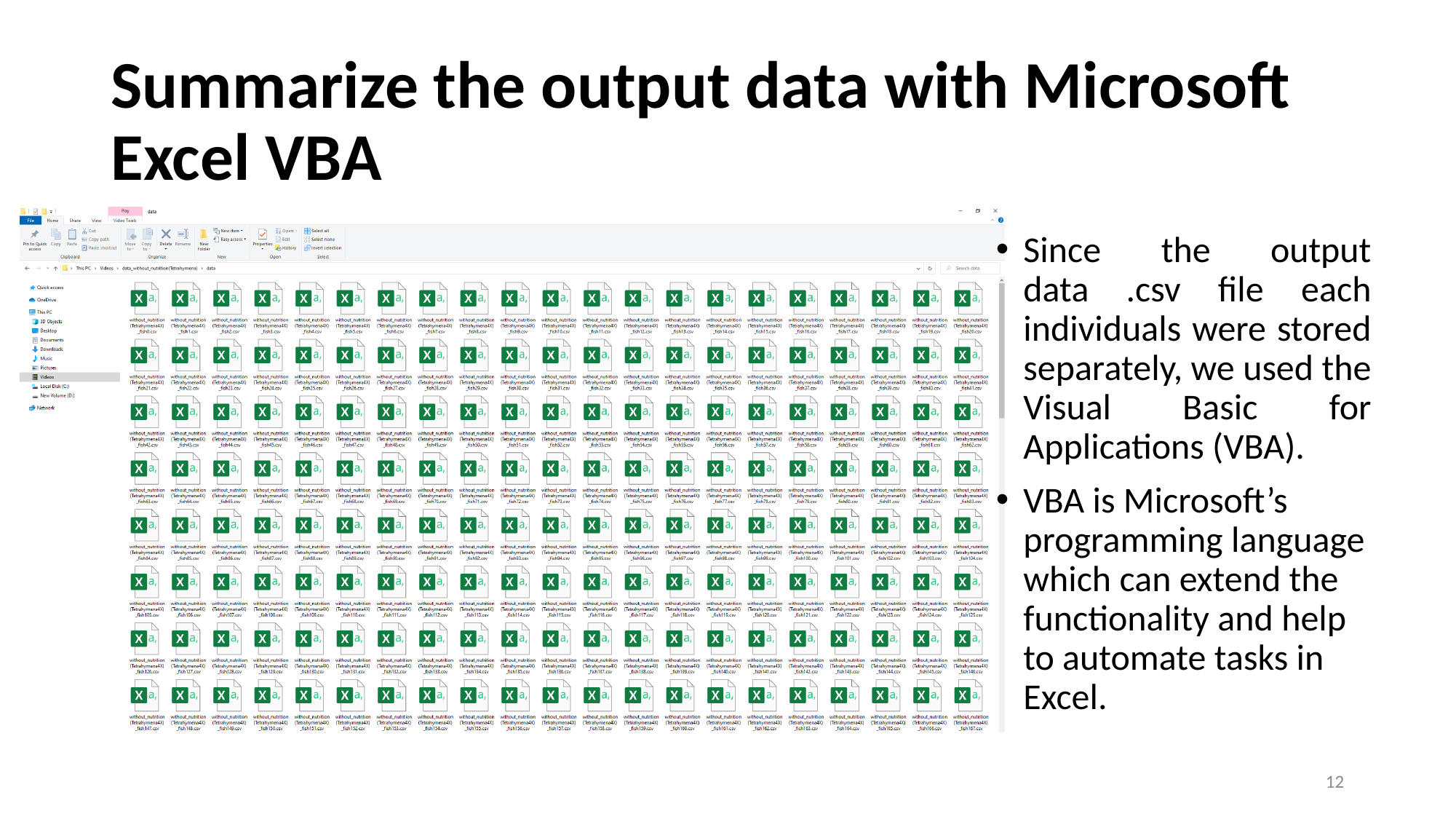

# Summarize the output data with Microsoft Excel VBA
Since the output data .csv file each individuals were stored separately, we used the Visual Basic for Applications (VBA).
VBA is Microsoft’s programming language which can extend the functionality and help to automate tasks in Excel.
12

## Slide 13
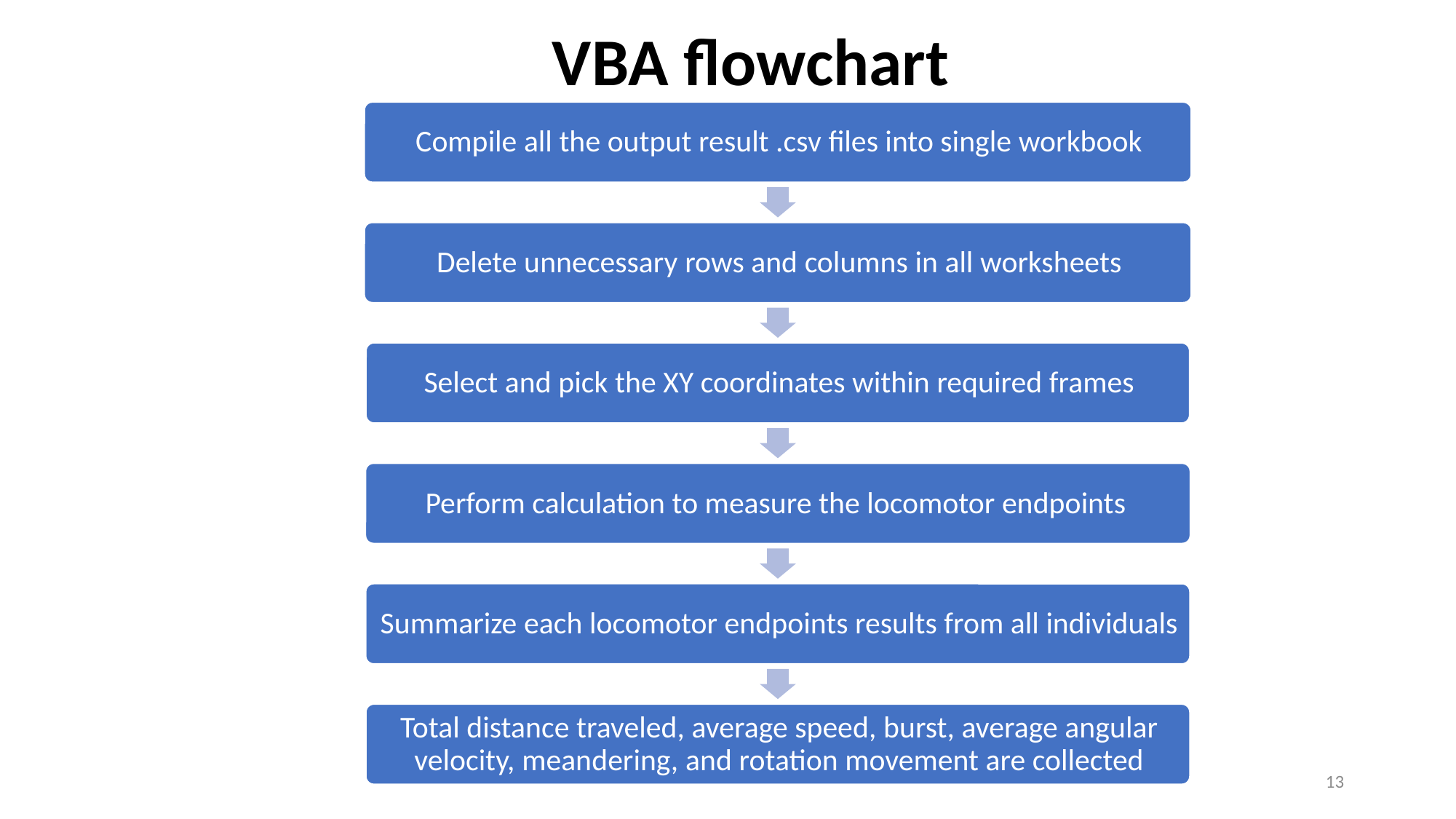

# VBA flowchart
13

## Slide 14
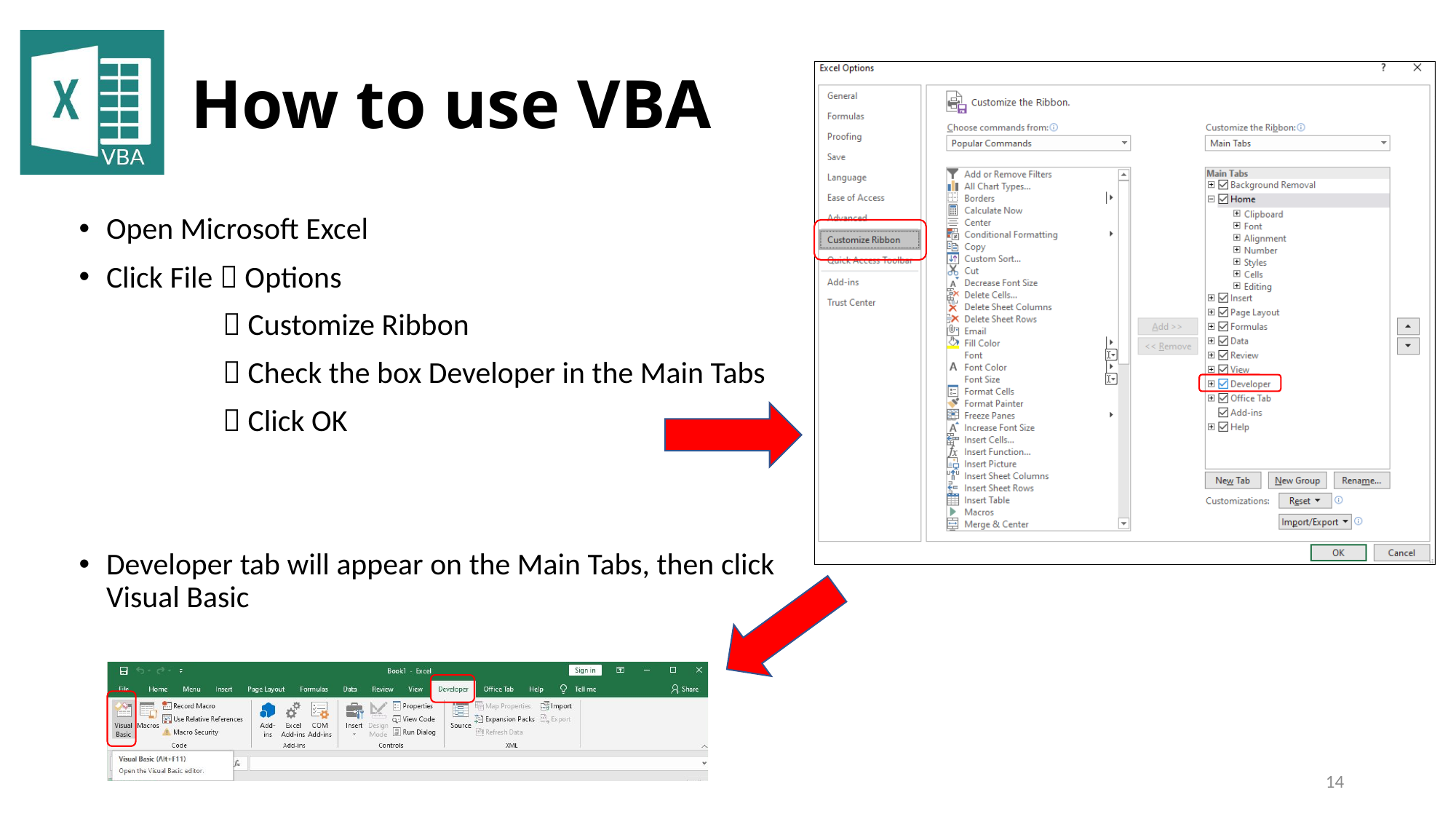

# How to use VBA
Open Microsoft Excel
Click File  Options
	  Customize Ribbon
	  Check the box Developer in the Main Tabs
	  Click OK
Developer tab will appear on the Main Tabs, then click Visual Basic
14

## Slide 15
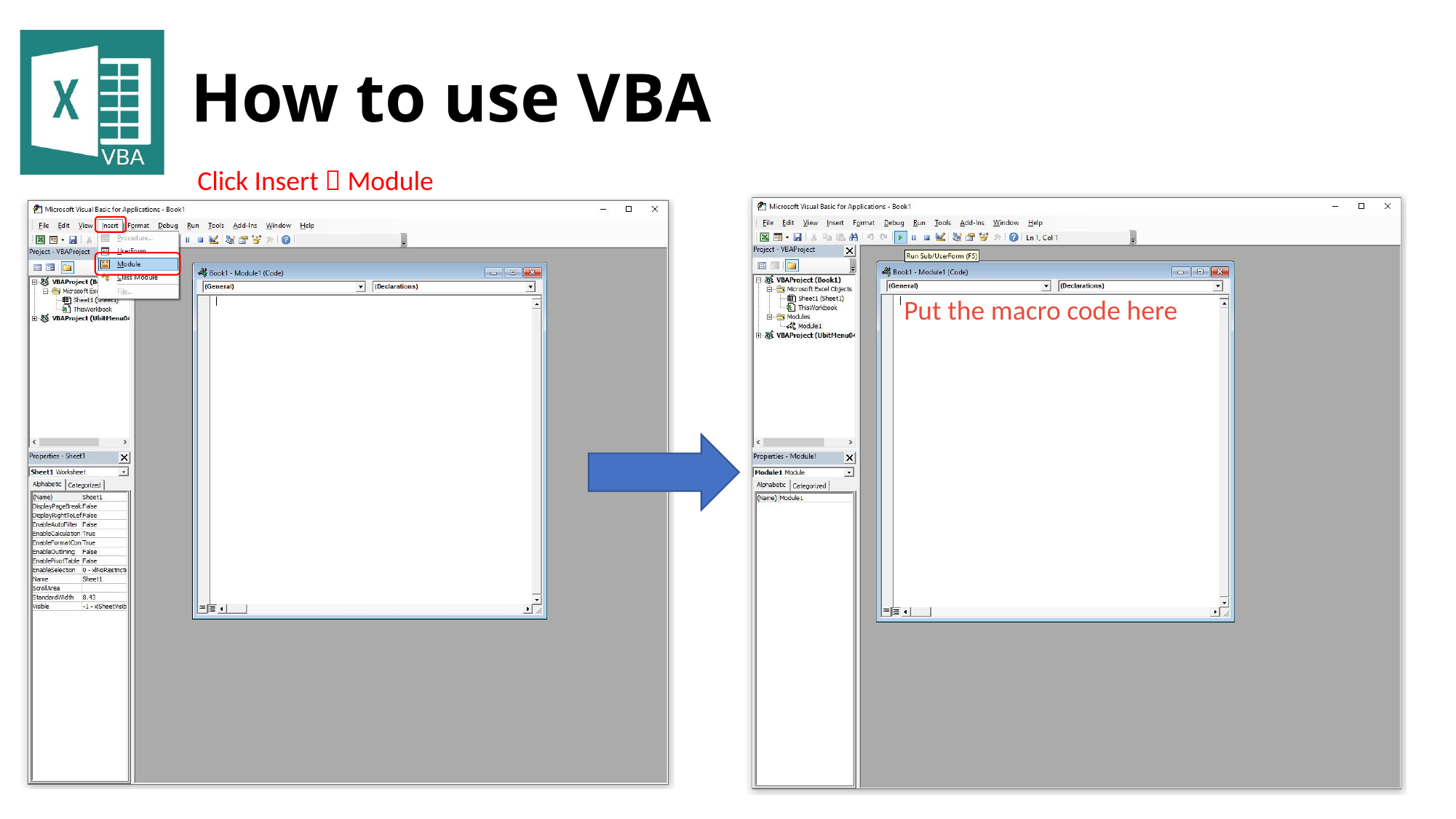

# How to use VBA
Click Insert  Module
Put the macro code here
15

## Slide 16
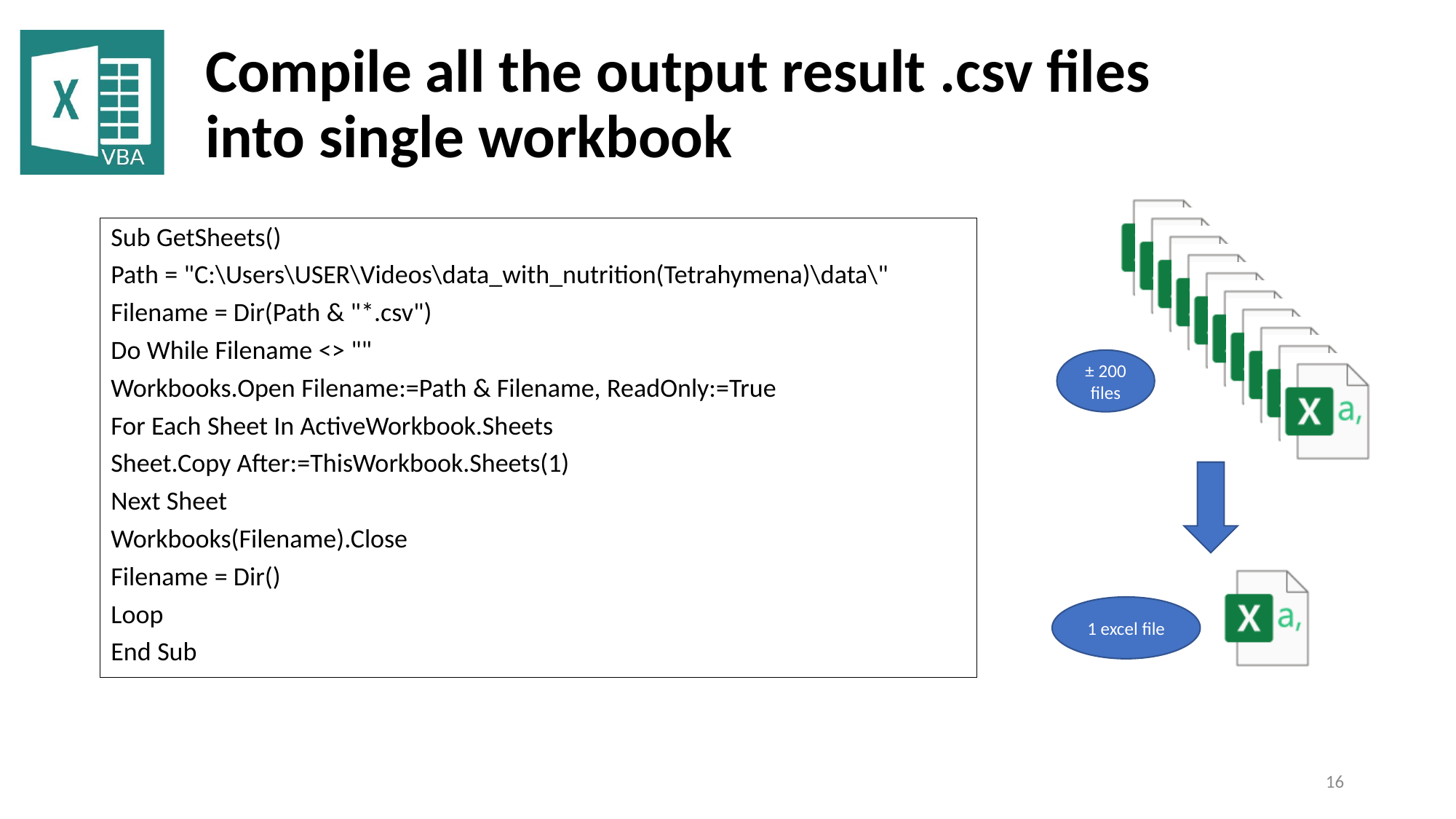

# Compile all the output result .csv files into single workbook
Sub GetSheets()
Path = "C:\Users\USER\Videos\data_with_nutrition(Tetrahymena)\data\"
Filename = Dir(Path & "*.csv")
Do While Filename <> ""
Workbooks.Open Filename:=Path & Filename, ReadOnly:=True
For Each Sheet In ActiveWorkbook.Sheets
Sheet.Copy After:=ThisWorkbook.Sheets(1)
Next Sheet
Workbooks(Filename).Close
Filename = Dir()
Loop
End Sub
± 200 files
1 excel file
16

## Slide 17
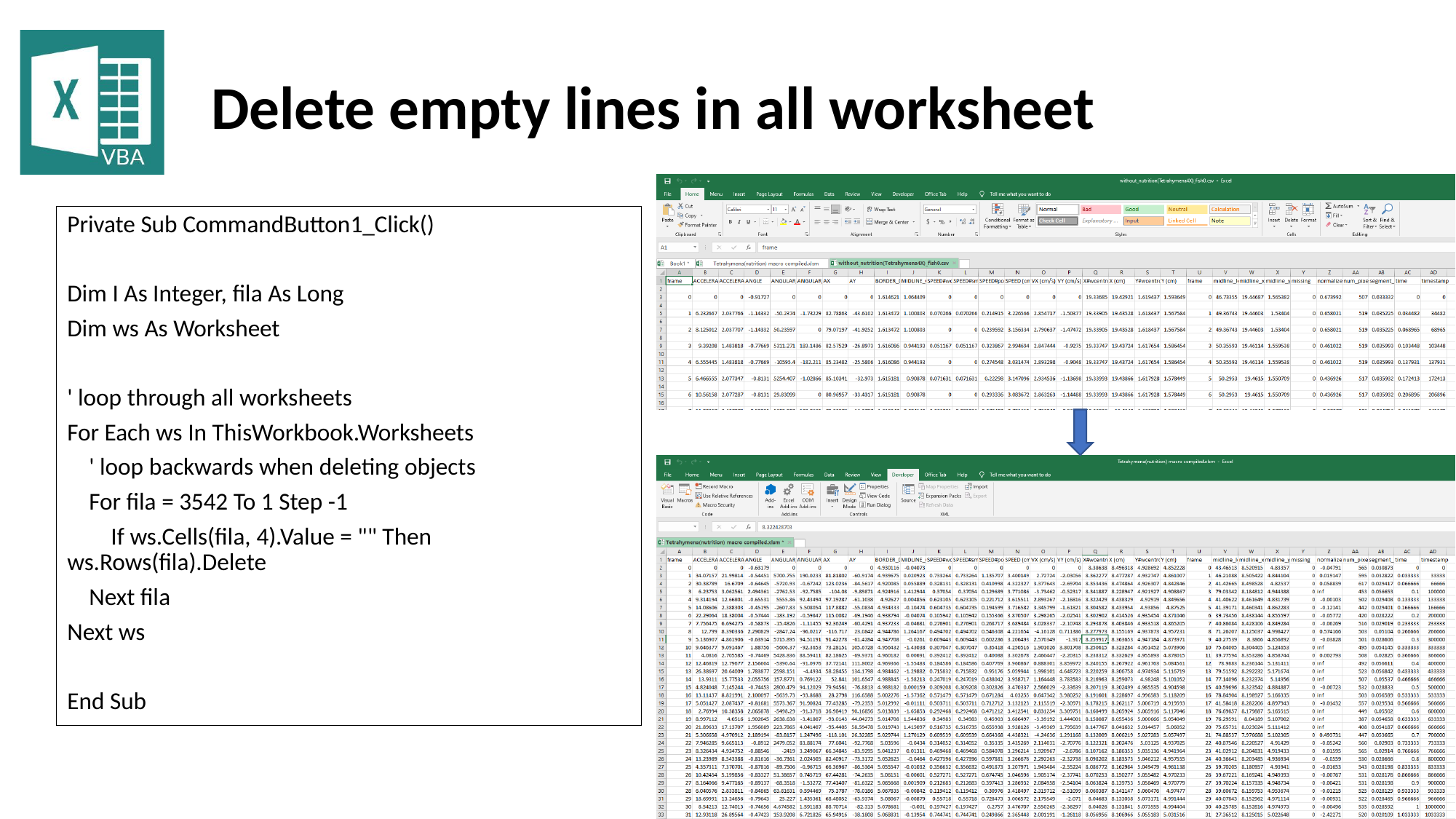

# Delete empty lines in all worksheet
Private Sub CommandButton1_Click()
Dim I As Integer, fila As Long
Dim ws As Worksheet
' loop through all worksheets
For Each ws In ThisWorkbook.Worksheets
 ' loop backwards when deleting objects
 For fila = 3542 To 1 Step -1
 If ws.Cells(fila, 4).Value = "" Then ws.Rows(fila).Delete
 Next fila
Next ws
End Sub
17

## Slide 18
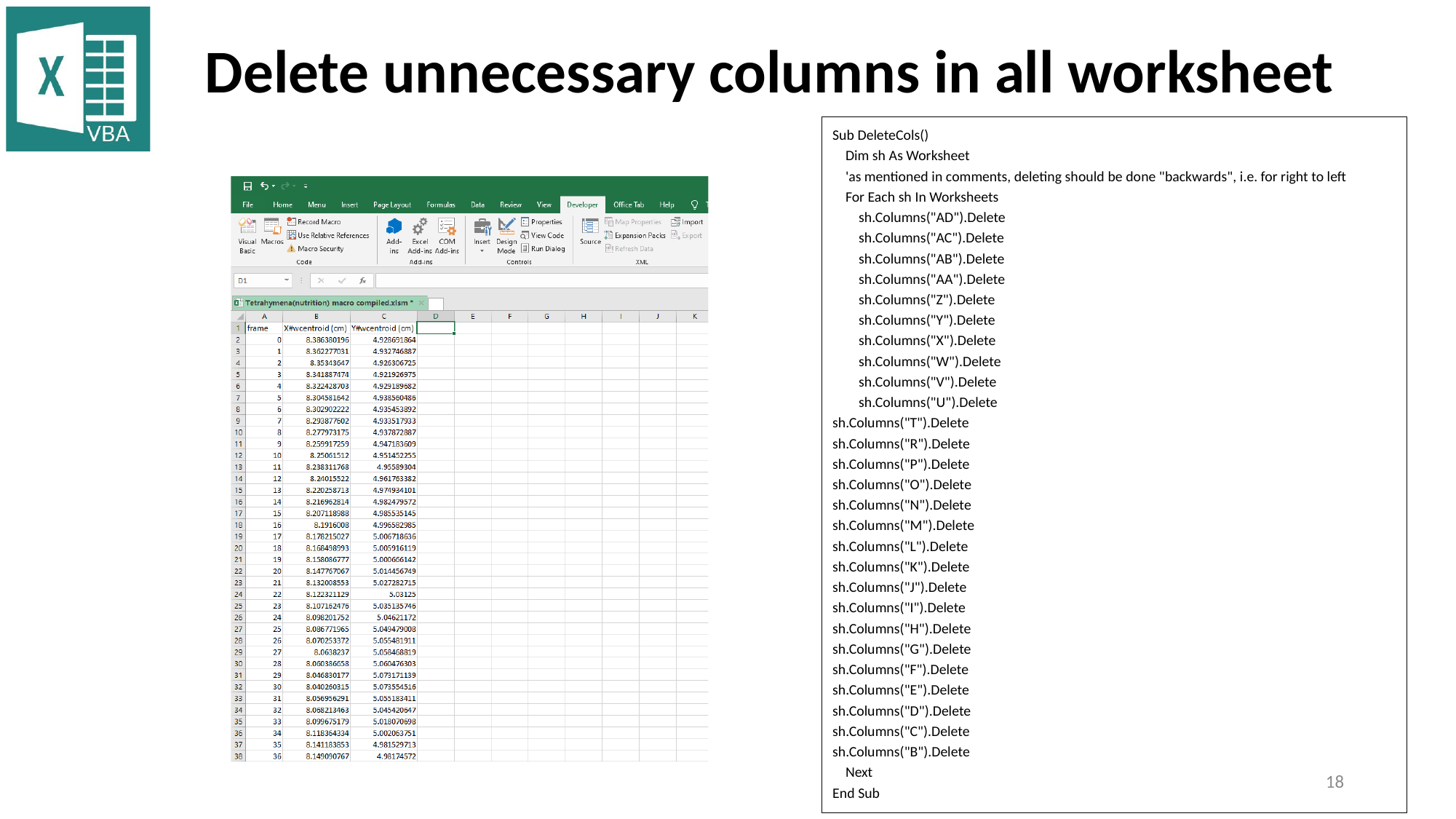

# Delete unnecessary columns in all worksheet
Sub DeleteCols()
 Dim sh As Worksheet
 'as mentioned in comments, deleting should be done "backwards", i.e. for right to left
 For Each sh In Worksheets
 sh.Columns("AD").Delete
 sh.Columns("AC").Delete
 sh.Columns("AB").Delete
 sh.Columns("AA").Delete
 sh.Columns("Z").Delete
 sh.Columns("Y").Delete
 sh.Columns("X").Delete
 sh.Columns("W").Delete
 sh.Columns("V").Delete
 sh.Columns("U").Delete
sh.Columns("T").Delete
sh.Columns("R").Delete
sh.Columns("P").Delete
sh.Columns("O").Delete
sh.Columns("N").Delete
sh.Columns("M").Delete
sh.Columns("L").Delete
sh.Columns("K").Delete
sh.Columns("J").Delete
sh.Columns("I").Delete
sh.Columns("H").Delete
sh.Columns("G").Delete
sh.Columns("F").Delete
sh.Columns("E").Delete
sh.Columns("D").Delete
sh.Columns("C").Delete
sh.Columns("B").Delete
 Next
End Sub
18

## Slide 19
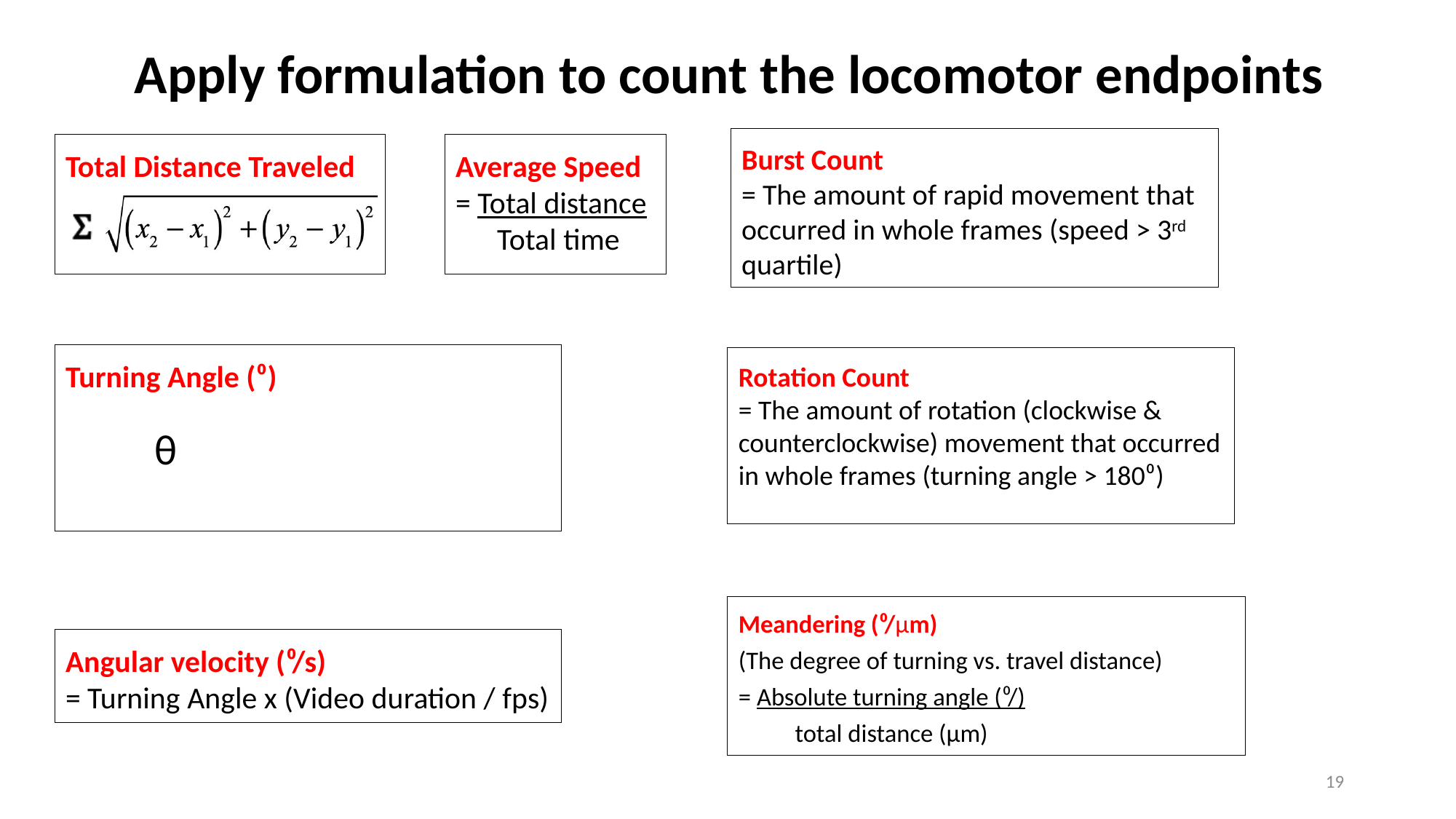

# Apply formulation to count the locomotor endpoints
Burst Count
= The amount of rapid movement that occurred in whole frames (speed > 3rd quartile)
Total Distance Traveled
Average Speed
= Total distance
 Total time
Turning Angle (⁰)
Rotation Count
= The amount of rotation (clockwise & counterclockwise) movement that occurred in whole frames (turning angle > 180⁰)
Meandering (⁰/µm)
(The degree of turning vs. travel distance)
= Absolute turning angle (⁰/)
 total distance (µm)
Angular velocity (⁰/s)
= Turning Angle x (Video duration / fps)
19

## Slide 20
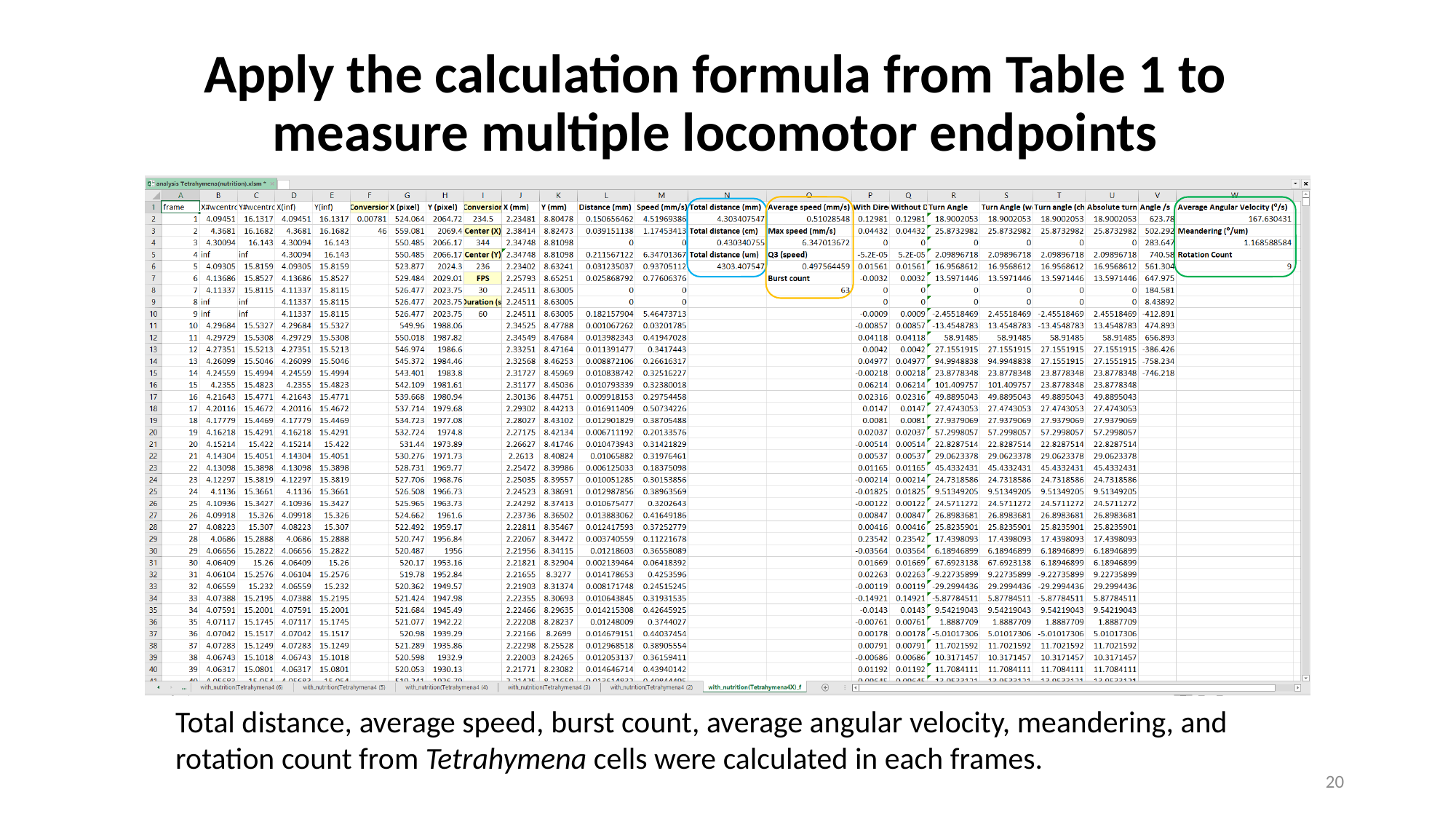

# Apply the calculation formula from Table 1 to measure multiple locomotor endpoints
Total distance, average speed, burst count, average angular velocity, meandering, and rotation count from Tetrahymena cells were calculated in each frames.
20

## Slide 21
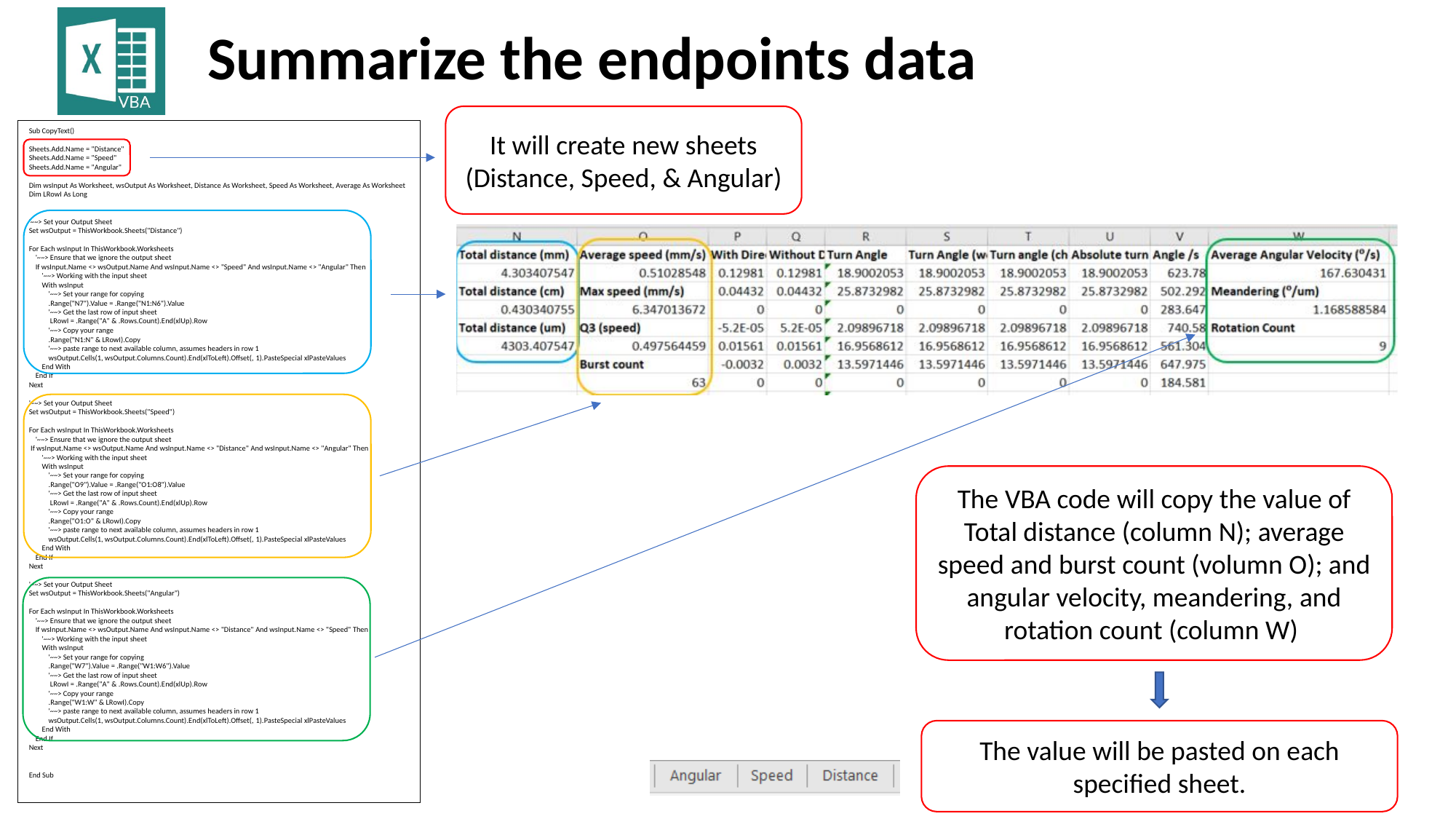

# Summarize the endpoints data
It will create new sheets
(Distance, Speed, & Angular)
Sub CopyText()
Sheets.Add.Name = "Distance"
Sheets.Add.Name = "Speed"
Sheets.Add.Name = "Angular"
Dim wsInput As Worksheet, wsOutput As Worksheet, Distance As Worksheet, Speed As Worksheet, Average As Worksheet
Dim LRowI As Long
'~~> Set your Output Sheet
Set wsOutput = ThisWorkbook.Sheets("Distance")
For Each wsInput In ThisWorkbook.Worksheets
 '~~> Ensure that we ignore the output sheet
 If wsInput.Name <> wsOutput.Name And wsInput.Name <> "Speed" And wsInput.Name <> "Angular" Then
 '~~> Working with the input sheet
 With wsInput
 '~~> Set your range for copying
 .Range("N7").Value = .Range("N1:N6").Value
 '~~> Get the last row of input sheet
 LRowI = .Range("A" & .Rows.Count).End(xlUp).Row
 '~~> Copy your range
 .Range("N1:N" & LRowI).Copy
 '~~> paste range to next available column, assumes headers in row 1
 wsOutput.Cells(1, wsOutput.Columns.Count).End(xlToLeft).Offset(, 1).PasteSpecial xlPasteValues
 End With
 End If
Next
'~~> Set your Output Sheet
Set wsOutput = ThisWorkbook.Sheets("Speed")
For Each wsInput In ThisWorkbook.Worksheets
 '~~> Ensure that we ignore the output sheet
 If wsInput.Name <> wsOutput.Name And wsInput.Name <> "Distance" And wsInput.Name <> "Angular" Then
 '~~> Working with the input sheet
 With wsInput
 '~~> Set your range for copying
 .Range("O9").Value = .Range("O1:O8").Value
 '~~> Get the last row of input sheet
 LRowI = .Range("A" & .Rows.Count).End(xlUp).Row
 '~~> Copy your range
 .Range("O1:O" & LRowI).Copy
 '~~> paste range to next available column, assumes headers in row 1
 wsOutput.Cells(1, wsOutput.Columns.Count).End(xlToLeft).Offset(, 1).PasteSpecial xlPasteValues
 End With
 End If
Next
'~~> Set your Output Sheet
Set wsOutput = ThisWorkbook.Sheets("Angular")
For Each wsInput In ThisWorkbook.Worksheets
 '~~> Ensure that we ignore the output sheet
 If wsInput.Name <> wsOutput.Name And wsInput.Name <> "Distance" And wsInput.Name <> "Speed" Then
 '~~> Working with the input sheet
 With wsInput
 '~~> Set your range for copying
 .Range("W7").Value = .Range("W1:W6").Value
 '~~> Get the last row of input sheet
 LRowI = .Range("A" & .Rows.Count).End(xlUp).Row
 '~~> Copy your range
 .Range("W1:W" & LRowI).Copy
 '~~> paste range to next available column, assumes headers in row 1
 wsOutput.Cells(1, wsOutput.Columns.Count).End(xlToLeft).Offset(, 1).PasteSpecial xlPasteValues
 End With
 End If
Next
End Sub
The VBA code will copy the value of Total distance (column N); average speed and burst count (volumn O); and angular velocity, meandering, and rotation count (column W)
The value will be pasted on each specified sheet.
21

## Slide 22
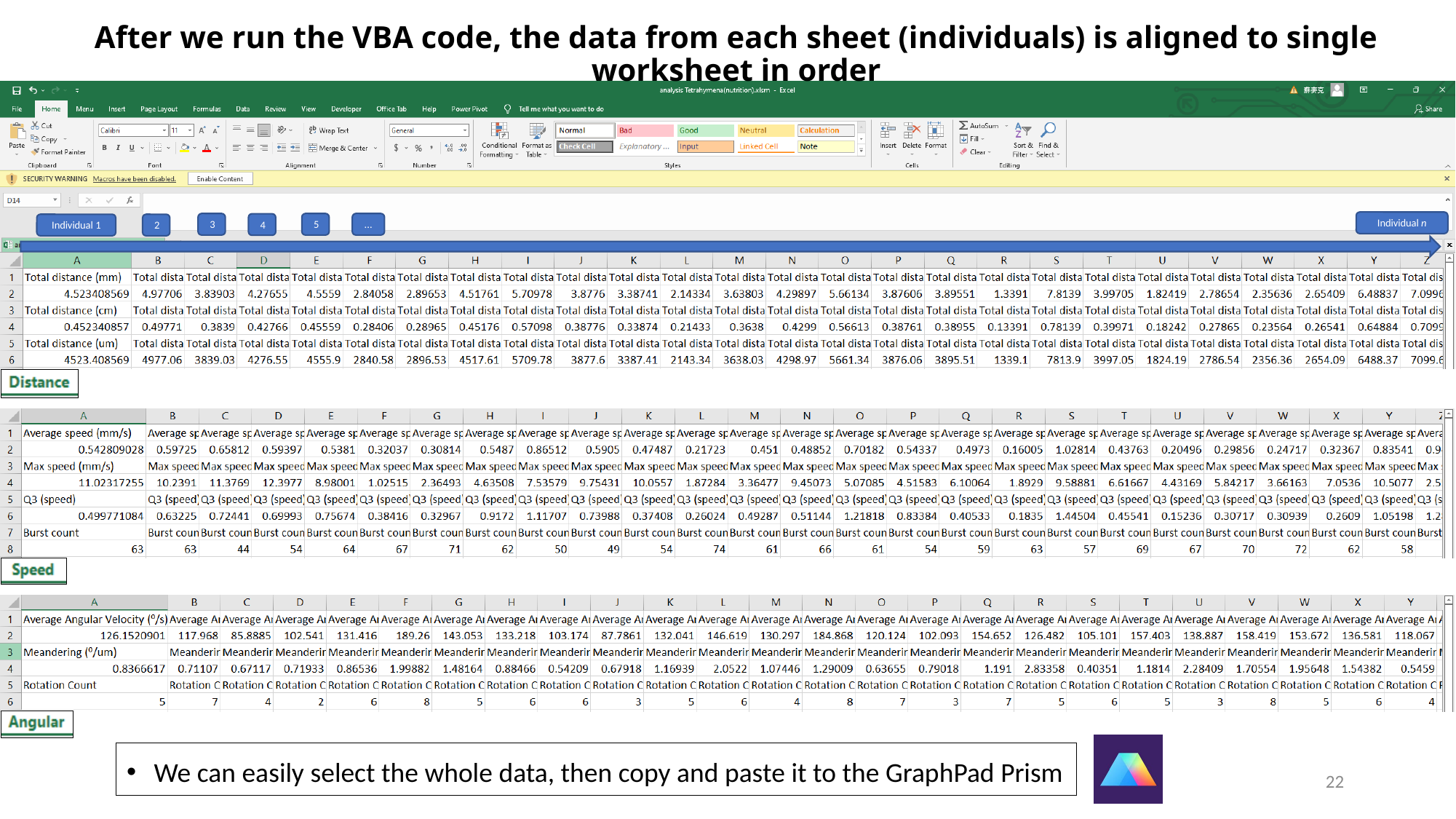

# After we run the VBA code, the data from each sheet (individuals) is aligned to single worksheet in order
Individual n
3
5
...
4
Individual 1
2
We can easily select the whole data, then copy and paste it to the GraphPad Prism
22

## Slide 23
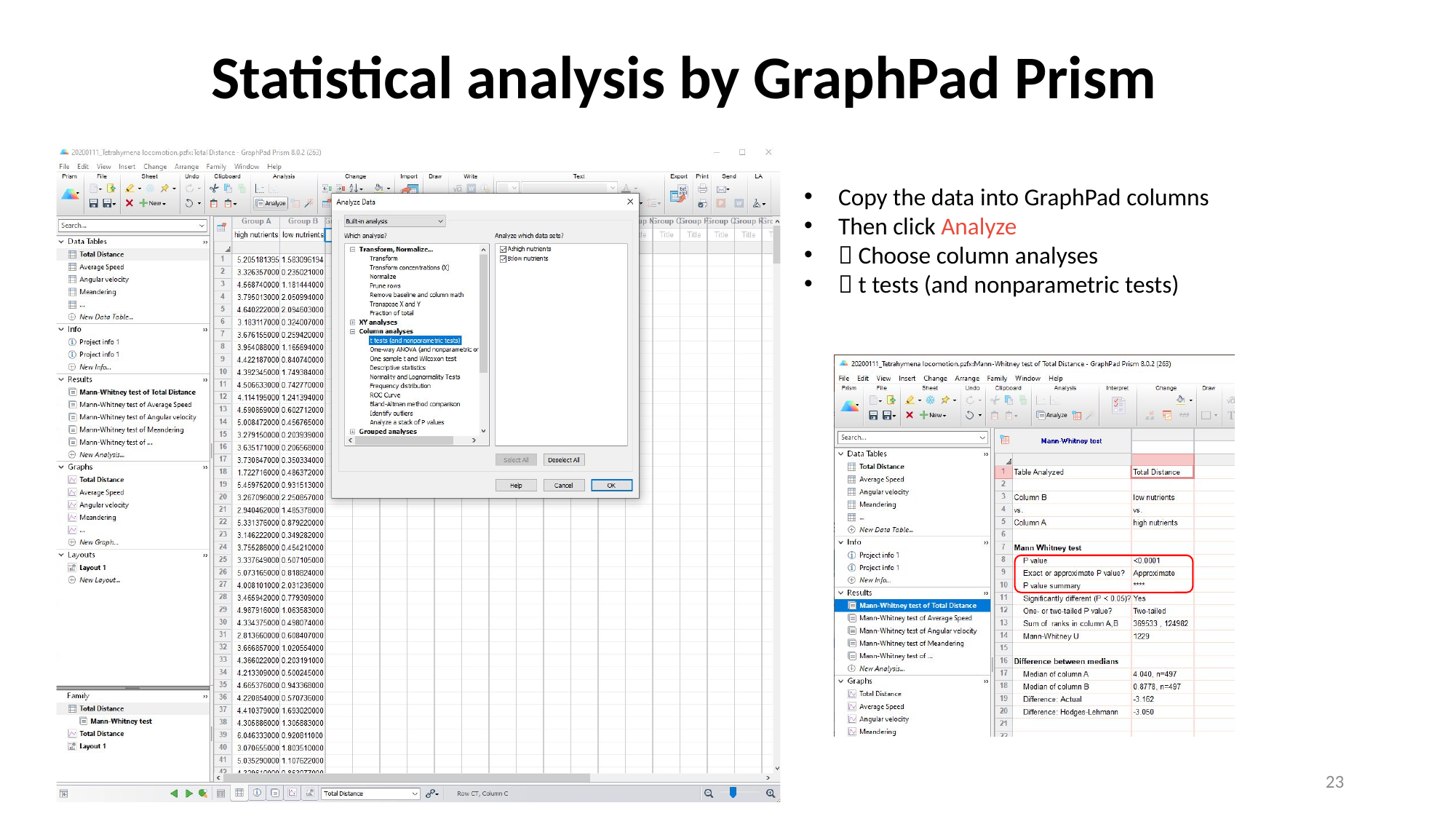

# Statistical analysis by GraphPad Prism
Copy the data into GraphPad columns
Then click Analyze
 Choose column analyses
 t tests (and nonparametric tests)
23

## Slide 24
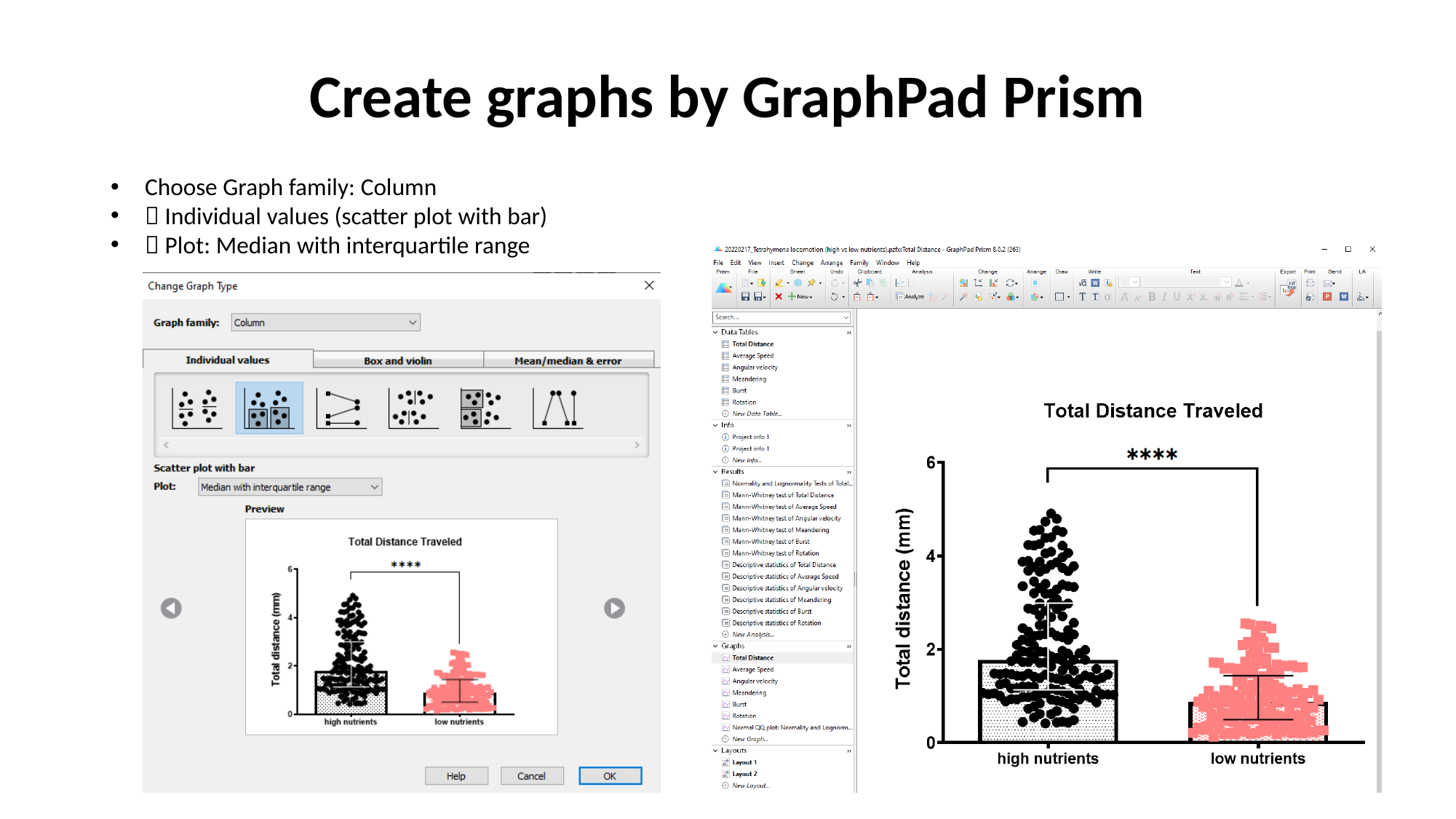

# Create graphs by GraphPad Prism
Choose Graph family: Column
 Individual values (scatter plot with bar)
 Plot: Median with interquartile range
24

## Slide 25
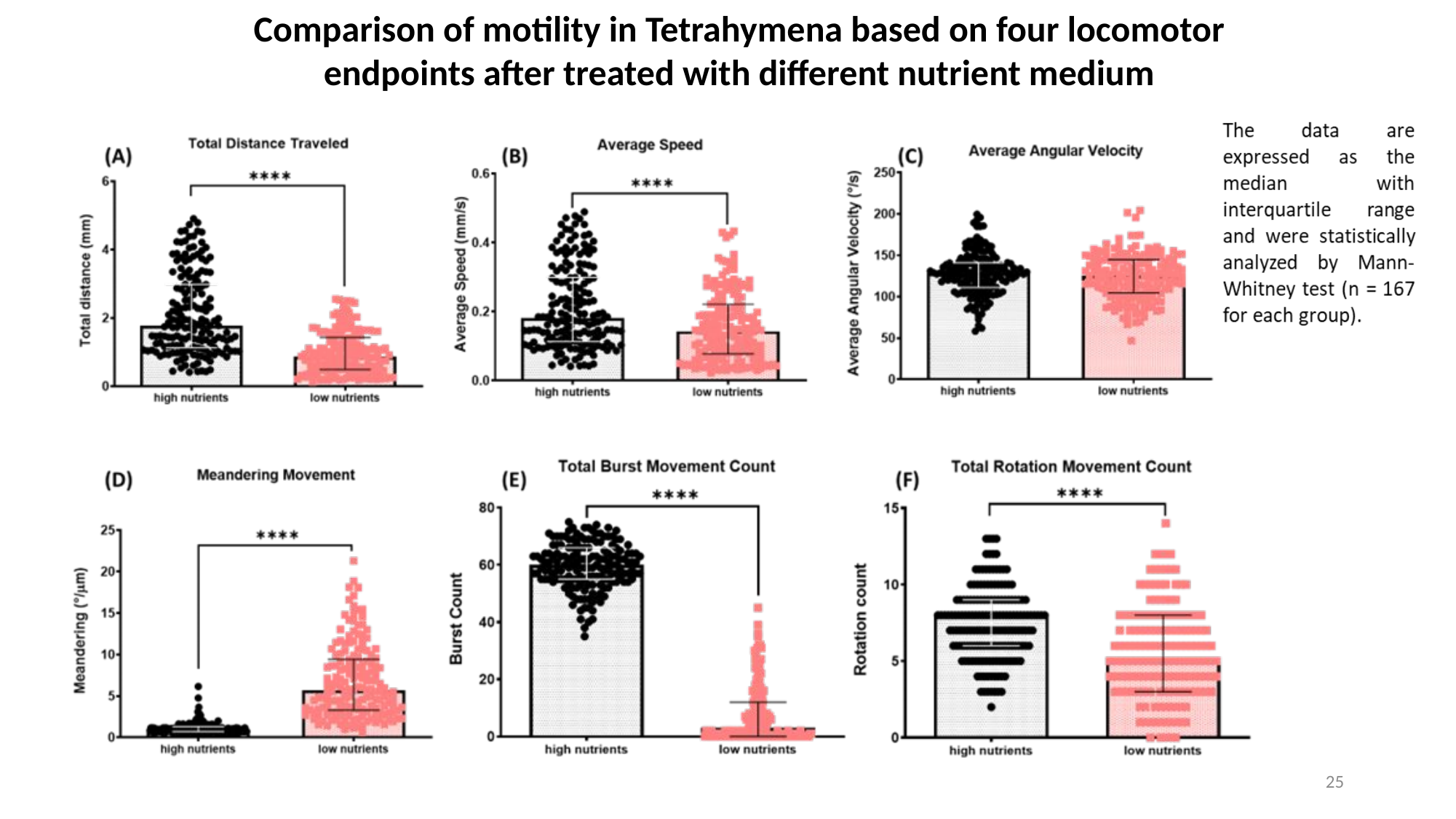

Comparison of motility in Tetrahymena based on four locomotor endpoints after treated with different nutrient medium
25

## Slide 26
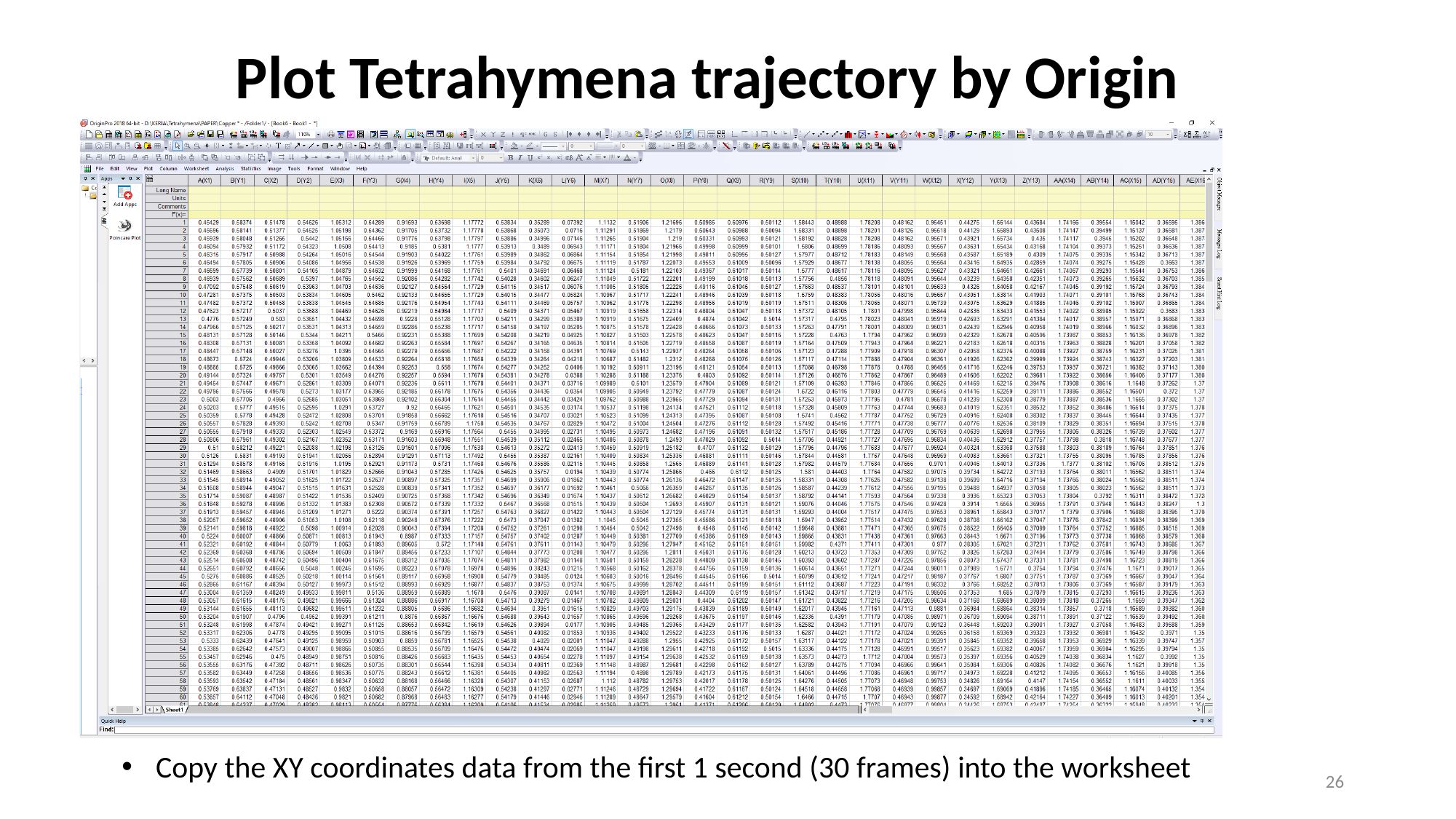

# Plot Tetrahymena trajectory by Origin
Copy the XY coordinates data from the first 1 second (30 frames) into the worksheet
26

## Slide 27
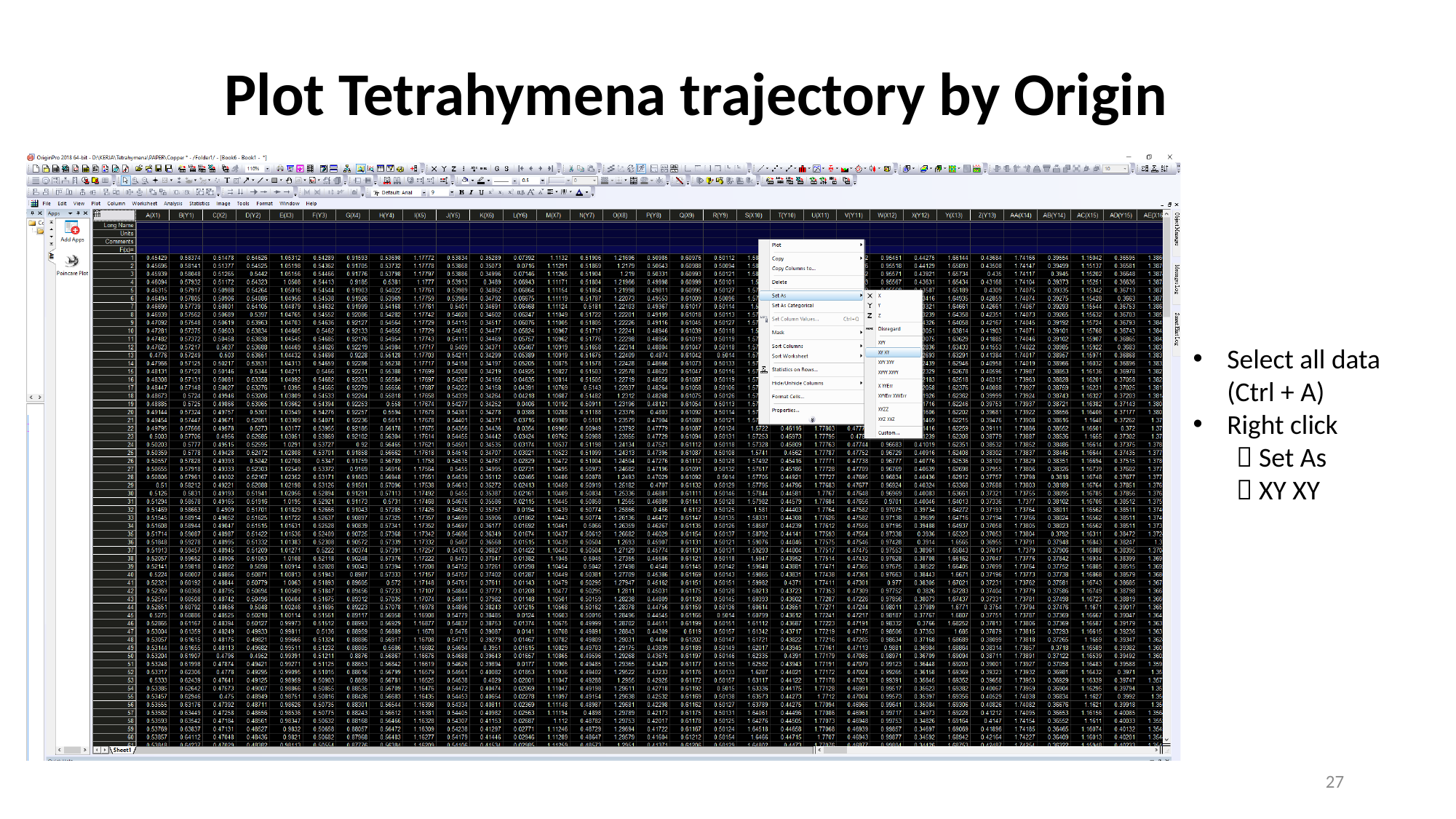

# Plot Tetrahymena trajectory by Origin
Select all data (Ctrl + A)
Right click
  Set As
  XY XY
27

## Slide 28
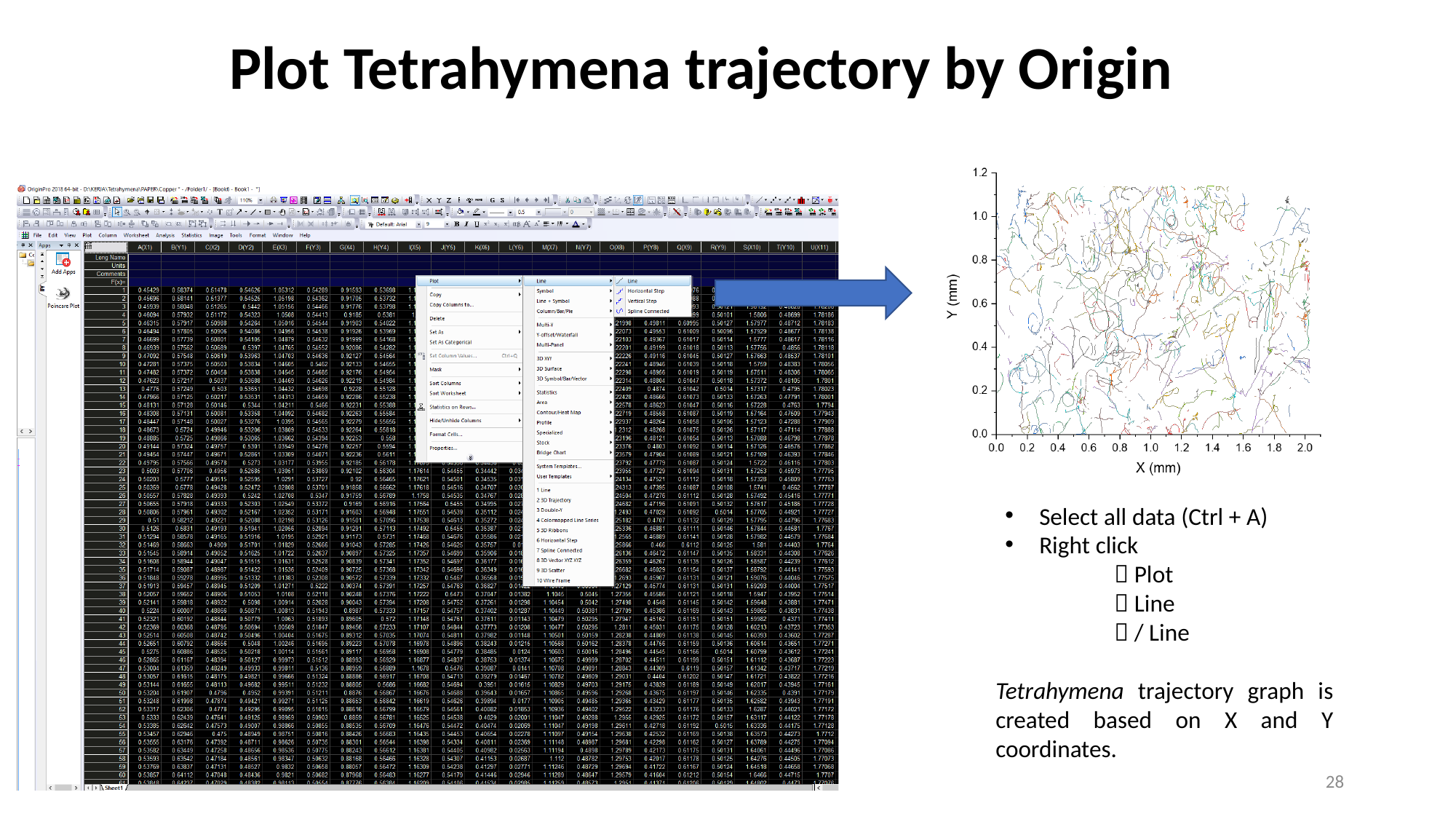

# Plot Tetrahymena trajectory by Origin
Select all data (Ctrl + A)
Right click
	 Plot
	 Line
	 / Line
Tetrahymena trajectory graph is created based on X and Y coordinates.
28

## Slide 29
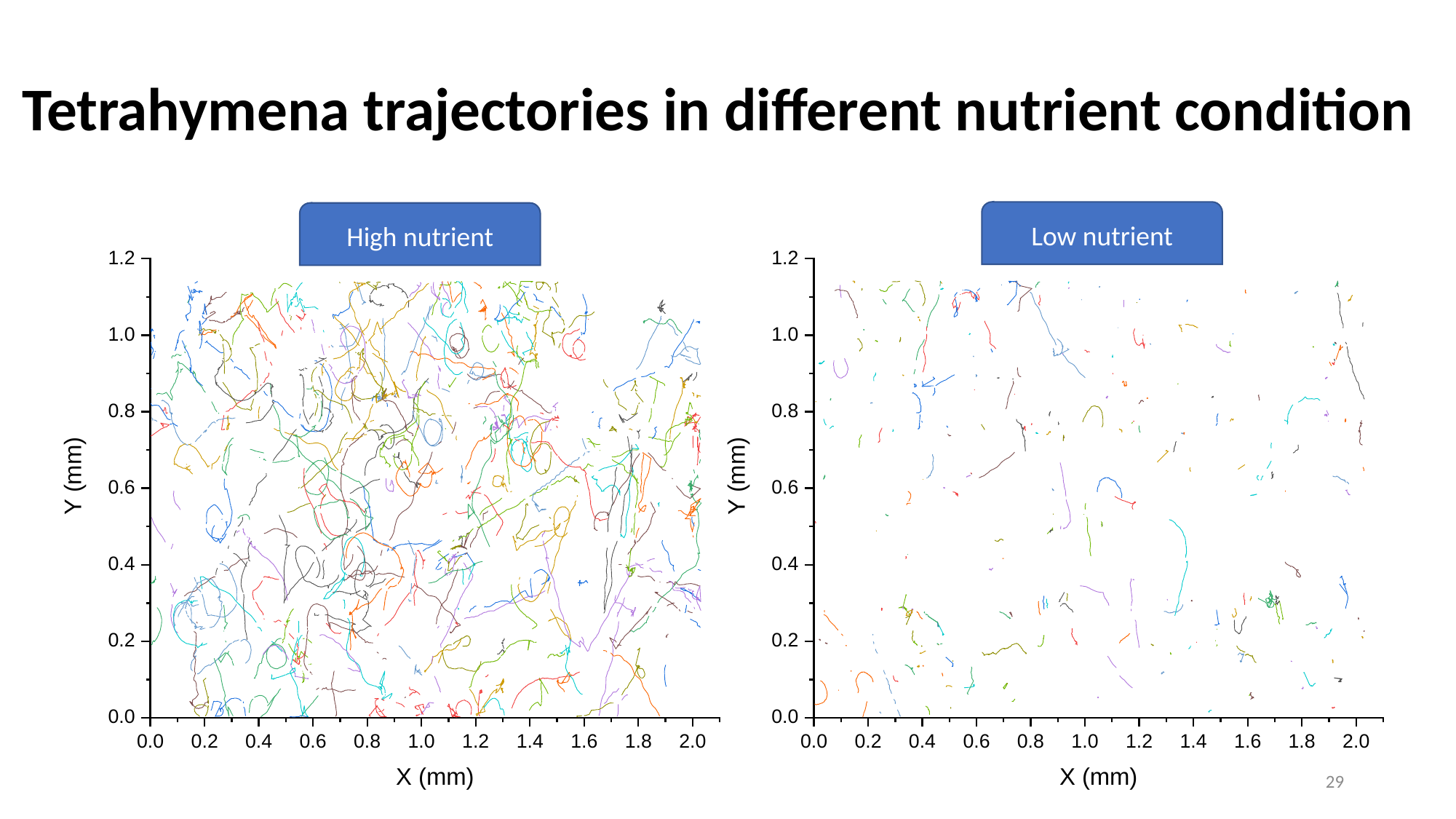

# Tetrahymena trajectories in different nutrient condition
Low nutrient
High nutrient
29
